# Wildlife Conservation on Private Land: A Social-Ecological Systems Study

**DOI:** 10.1007/s00267-024-01962-w

**Published:** 2024-03-23

**Authors:** Matthew Taylor, Barry Brook, Christopher Johnson, Siobhan de Little

**Affiliations:** 1 College of Sciences and Engineering, University of Tasmania, Hobart, TAS Australia; 2 Ecotec Environmental, Brunswick, VIC Australia

**Keywords:** Wildlife conservation, Social-ecological systems, Private land, Transdisciplinary research, Citizen science

## Abstract

As human activity accelerates the global crisis facing wildlife populations, private land conservation provides an example of wildlife management challenges in social-ecological systems. This study reports on the research phase of ‘WildTracker’ - a co-created citizen science project, involving 160 landholders across three Tasmanian regions. This was a transdisciplinary collaboration between an environmental organisation, university researchers, and local landholders. Focusing on mammal and bird species, the project integrated diverse data types and technologies: social surveys, quantitative ecology, motion sensor cameras, acoustic recorders, and advanced machine-learning analytics. An iterative analytical methodology encompassed Pearson and point-biserial correlation for interrelationships, Non-Metric Multidimensional Scaling (NMDS) for clustering, and Random Forest machine learning for variable importance and prediction. Taken together, these analyses revealed complex relationships between wildlife populations and a suite of ecological, socio-economic, and land management variables. Both site-scale habitat characteristics and landscape-scale vegetation patterns were useful predictors of mammal and bird activity, but these relationships were different for mammals and birds. Four focal mammal species showed variation in their response to ecological and land management drivers. Unexpectedly, threatened species, such as the eastern quoll (*Dasyurus viverrinus)*, favoured locations where habitat was substantially modified by human activities. The research provides actionable insights for landowners, and highlights the importance of ‘messy,’ ecologically heterogeneous, mixed agricultural landscapes for wildlife conservation. The identification of thresholds in habitat fragmentation reinforced the importance of collaboration across private landscapes. Participatory research models such as WildTracker can complement efforts to address the wicked problem of wildlife conservation in the Anthropocene.

## Introduction

### Context and Importance of Wildlife Conservation in the Anthropocene

Human land use has precipitated a major global decline in biodiversity, especially on private lands. Vertebrate populations declined by 69% between 1970 and 2018, and habitat conversion on private lands was a significant driver of these declines (Almond et al. 2022). A high proportion of Australia’s species are endemic and much of its unique biodiversity is found on private lands. The continent’s rich biodiversity is therefore both threatened and protected by actions on private properties (Fitzsimons 2015; Legge et al. 2023). Private lands play a crucial role in global biodiversity conservation (Knight 1999), where they harbour rich and unique ecosystems. Private land ownership was historically focused on productive parts of the landscape and private properties continue to support abundant and diverse wildlife communities, including threatened species (Rayner et al. 2014; Jenkins et al. 2015; Clancy et al. 2020).

Private lands present conservation opportunities and challenges distinct from public reserves, such as aligning landowner interests with broader ecological goals and overcoming knowledge gaps (Ivanova and Cook 2020; Bingham et al. 2021). Wildlife populations do not conform to human-designated boundaries, making their management on private lands inherently complex (Pulsford et al. 2013). The distribution, movement, and life-history strategies of species necessitate conservation approaches that are spatially explicit, accounting for both site-scale habitat requirements and landscape-level processes that facilitate migration corridors, habitat connectivity, and ecological fluxes across multiple properties (Mackey et al. 2013).

Private lands also offer unique opportunities for conservation. With appropriate management, these lands can serve as vital refuges and ‘stepping stones’ for wildlife between protected areas and reserves, mitigating some of the impacts of habitat fragmentation (Figgis 2004; Fitzsimons 2004; Kamal et al. 2015; Chapman et al. 2023). Conservation covenant (easement) and stewardship programs, alongside innovative approaches like citizen science and participatory action research, can complement public reserve systems and contribute to multi-tenure conservation networks (Pulsford et al. 2013; Kamal et al. 2014; Taylor et al. 2023a). Private land conservation strategies engage landholders directly in conservation efforts, fostering knowledge sharing and collaborative management practices. This grassroots involvement is vital for effective stewardship of private lands and contributes significantly to global biodiversity conservation efforts.

### The Social Ecological Systems Framework

Wildlife conservation on private lands is complex because it lies at the intersection of ecology, economics, and human values, making it a quintessential example of a “wicked problem” (Rittel and Webber 1973). Wicked problems are characterized by the lack of clear definitions, solutions, or objective measures of success, and they typically encompass various intertwined and often conflicting human and ecological dimensions. Social-ecological perspectives are vital in addressing such wicked problems, as they emphasize the interconnectedness of human and environmental systems (Ostrom 2009; Mertens 2015; Akamani et al. 2016). This approach recognises that conservation outcomes are influenced not just by ecological factors, but also by social, economic, and cultural dynamics. On private lands, where decisions of individual landowner can have major effects on conservation efforts, gaining a better understanding of these interdependencies in the context of wildlife management is crucial. Adopting a social-ecological perspective allows for more holistic and effective strategies, as it integrates diverse stakeholder values, knowledge systems, and ecological processes, leading to more sustainable and community-supported conservation outcomes (Angelstam et al. 2013; Hummel et al. 2017; Hull et al. 2023). The application of this approach to wildlife management is a potential pathway to better understanding and addressing wicked problems that have to date largely defied resolution, despite significant research and management effort globally.

Transdisciplinary research, which transcends disciplinary boundaries and incorporates knowledge from both scientific and non-scientific sources, is increasingly recognised as a valuable approach for investigating social-ecological systems (Axelsson 2012). By involving multiple stakeholders, including local landowners, ecologists, policymakers, and the broader community, transdisciplinary research fosters holistic understandings and collaborative strategies for environmental management (Marchini et al. 2021). Co-created citizen science projects are an example of transdisciplinary research, which offers powerful tool to bridge knowledge gaps, by harnessing the collective power of the community in monitoring and understanding the environment (Bonney et al. 2009; Crain et al. 2014; Strasser et al. 2019). Citizen science enables researchers to gather data at scales previously unattainable, while participants benefit from enhanced environmental awareness and a sense of stewardship. Co-created knowledge can also be used to inform and thereby improve landholders’ management of their land (Toomey and Domroese 2013; Taylor et al. 2023b). More than just a data collection tool, citizen science fosters collaborations that can inform sustainable land-management practices and empower local communities to take active roles in conservation efforts, ultimately contributing to more robust environmental outcomes (Dickinson et al. 2010; Conrad and Hilchey 2011; Tulloch et al. 2013).

### Research Gap and Objectives of the Study

Although the role of private land in wildlife conservation has been repeatedly acknowledged (Knight 1999, Fitzsimons 2015, Bingham et al. 2021), comprehensive social-ecological studies that integrate socio-economic, ecological, and land management variables at various spatial scales are lacking. Tasmania is a large (68,000 km2) temperate island off the south coast of Australia. Tasmania’s diverse range of ecosystems and species, including many that are threatened and endemic, makes it a microcosm for understanding broader global patterns in wildlife conservation on ecologically heterogeneous, human-dominated private landscapes. The region’s diverse land uses and mix of private and public lands, coupled with active community involvement in land management, makes Tasmania a relevant case study that resonates with global social-ecological research into wildlife management.

In this study, we adopted a collaborative transdisciplinary approach, prioritising co-design with participants over traditional hypothesis development. Therefore, our preliminary research objectives were deliberately general to allow for input from landholders, practitioners, and researchers. This was achieved through a co-design workshop process. The preliminary research objectives were as follows:

1. Explore the relationships between wildlife populations and a variety of site and landscape variables on private lands.
2. Employ a transdisciplinary approach, integrating ecological data with socio-economic and land management factors.
3. Use appropriately sophisticated analytical methods to robustly discern patterns and drivers affecting wildlife on private properties.
4. Offer practical insights for landowners and conservationists, aiming to promote collaborative, landscape-scale conservation efforts.

Our approach to hypothesis formulation is detailed in the methodology section. This process was essential in fostering a participatory research environment. The objectives served as a guiding framework, setting the scope without constraining specific investigative paths. The stakeholder co-design process culminated in the formulation of a series of interrelated hypotheses, embodied in a conceptual model that reflects the dynamics of the social-ecological system (Fig. 2). The linkages between nodes in our conceptual model represent hypotheses regarding relationships between those nodes, such as between land management practices and ecological pressures, or between ecological pressures and wildlife outcomes for mammals, birds, or focal species. These hypotheses were subsequently tested through a comprehensive suite of social and ecological data-collection and analytical methods, ensuring a robust yet inclusive exploration of the system’s complexities.

### Focal Wildlife Groups and Taxa

This study concentrated on native mammals and bird diversity, with a detailed focus on four mammal species. The overarching objective was to compare the biotic responses to landscape and site-scale socio-ecological drivers, a phenomenon under-explored in existing literature. While previous comparative studies have indicated that mammals and birds are similarly impacted by intensive land use, they also show nuanced differences in their reactions to landscapes that are less-intensively modified (Burel et al. 1998; Felton et al. 2010; Santangeli et al. 2022). Moreover, mammals and birds, being charismatic and readily identifiable, are of particular community interest, and established survey procedures are suitable for deployment by citizen scientists. The selection of the four focal species (eastern barred bandicoot (*Perameles gunnii*), eastern bettong (*Bettongia gaimardi*), eastern quoll (*Dasyurus viverrinus*) and long-nosed potoroo (*Potorous tridactylus*)) for detailed analysis of social-ecological drivers was guided by the outputs of the stakeholder workshop and the specific interests expressed by landholders. The species selected are all in the ‘critical weight range’ of high extinction risk in Australia (Johnson and Isaac 2009) and are especially susceptible to predation by feral cats. The species were expected to show both commonalities and contrasts in their response to habitat condition at site and landscape scales, thereby providing valuable insights into the complex dynamics of habitat-species interactions and socio-ecological drivers.

## Methods

**Fig. 1:**
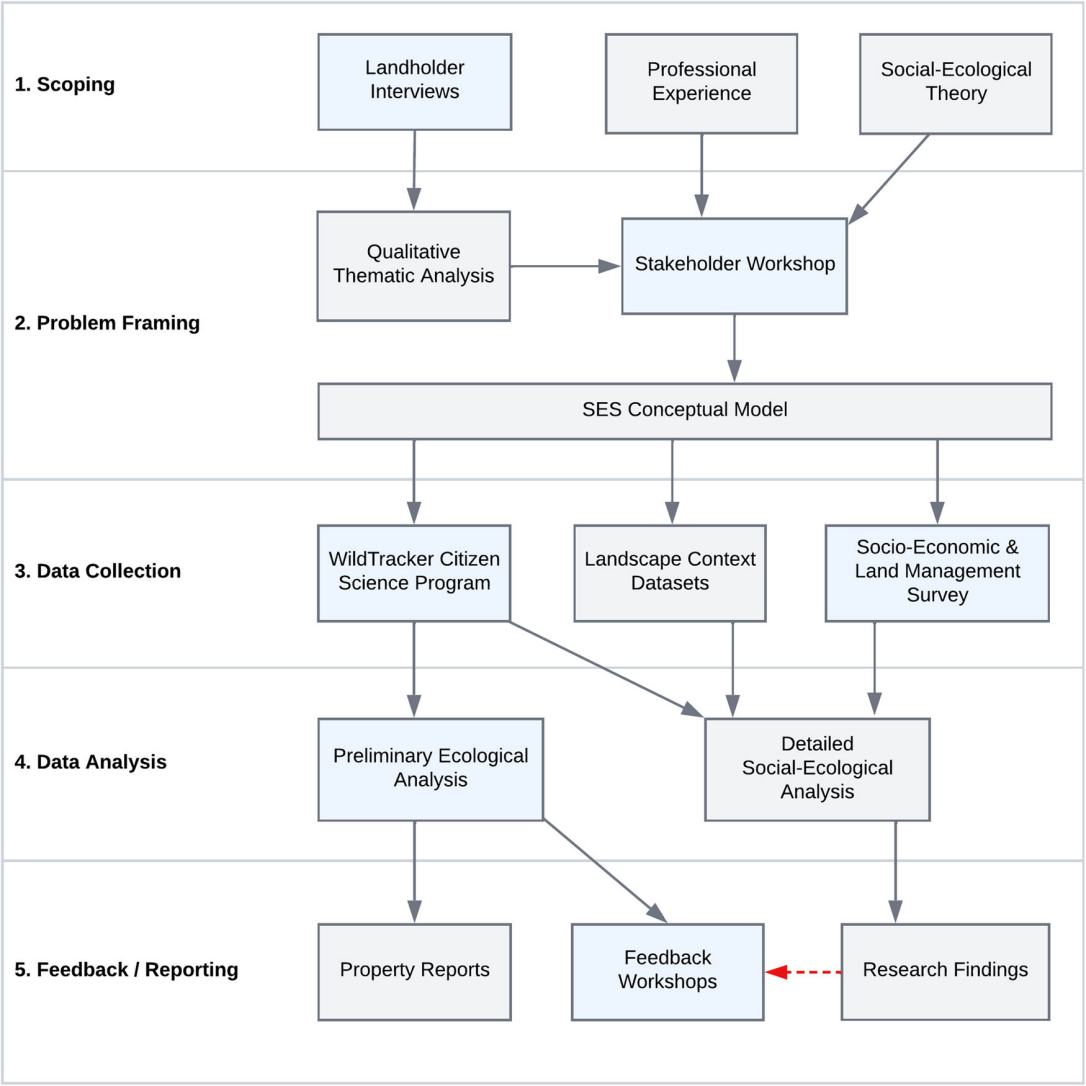
Diagram of the five-stage social-ecological research methodology. Blue shaded elements indicate landholder participation. Preliminary research findings were presented to landholders at feedback workshops to promote discussion. However, logistical complications delayed final analyses and final results were presented to landholders via a project report after the workshops, and via tailored property reports provided to each landholder

This research adopted a transdisciplinary methodology grounded in the principles of citizen science and participatory action research. Social-ecological systems are intricate, with intertwined human and natural components that influence one another in complex ways. In order to understand these systems a combination of quantitative and qualitative methods was used, drawing from both the natural and social sciences. The research process involved private landholders in five stages, including scoping, problem framing, data collection, data analysis, and feedback and reporting. Conservation practitioners and researchers were also involved during the scoping and problem framing stages of the research. The data collection and analysis procedures are illustrated in Fig. 1. Stakeholder involvement in scoping and problem framing are essential elements of participatory research that ensure relevance by incorporating local ideas and knowledge systems into both research planning and implementation.

## Co-Design Framework

**Fig. 2:**
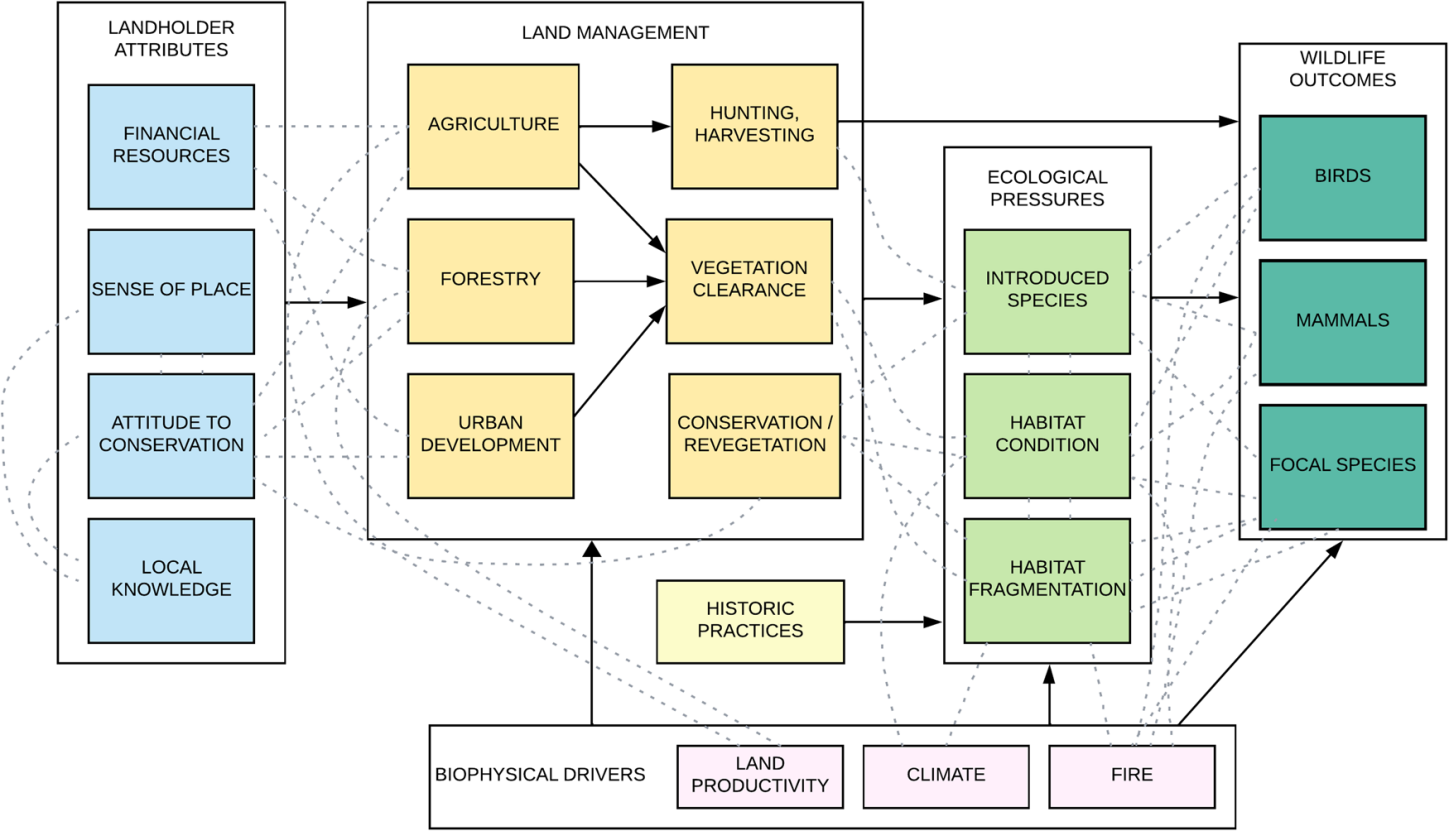
Social-ecological systems model of wildlife conservation on private lands, developed by landholders, conservation practitioners and researchers. The model represents relationships between high-level social-ecological factors, and guided the data collection and analysis processes. Major relationships between model elements are represented by black arrows. Note that fire and climate drivers were not incorporated into data aggregation or analysis. The co-design workshop process is described in detail in Taylor et al. (2023b)

The scoping and problem framing stages of the research are covered in detail in previously published papers (Taylor et al. 2023a, 2023b). A social-ecological systems conceptual model (Fig. 2) was developed through a stakeholder workshop involving a group of 21 participants, comprising landholders (*N* = 8), conservation practitioners (*n* = 6), and researchers (*n* = 7). Workshop participants identified the most significant social-ecological drivers of wildlife conservation and a conceptual model was produced that represented causal relationships between model elements. The conceptual model can be thought of as a set of interrelated hypotheses regarding the relationships between landholders, their management practices, ecological pressures, biophysical drivers, and wildlife conservation outcomes. The model guided the data-collection phase of the research, with data gathered on socio-ecological variables (model elements). Our iterative approach to testing these hypotheses is explained in the methods section.

### Study Location

**Fig. 3:**
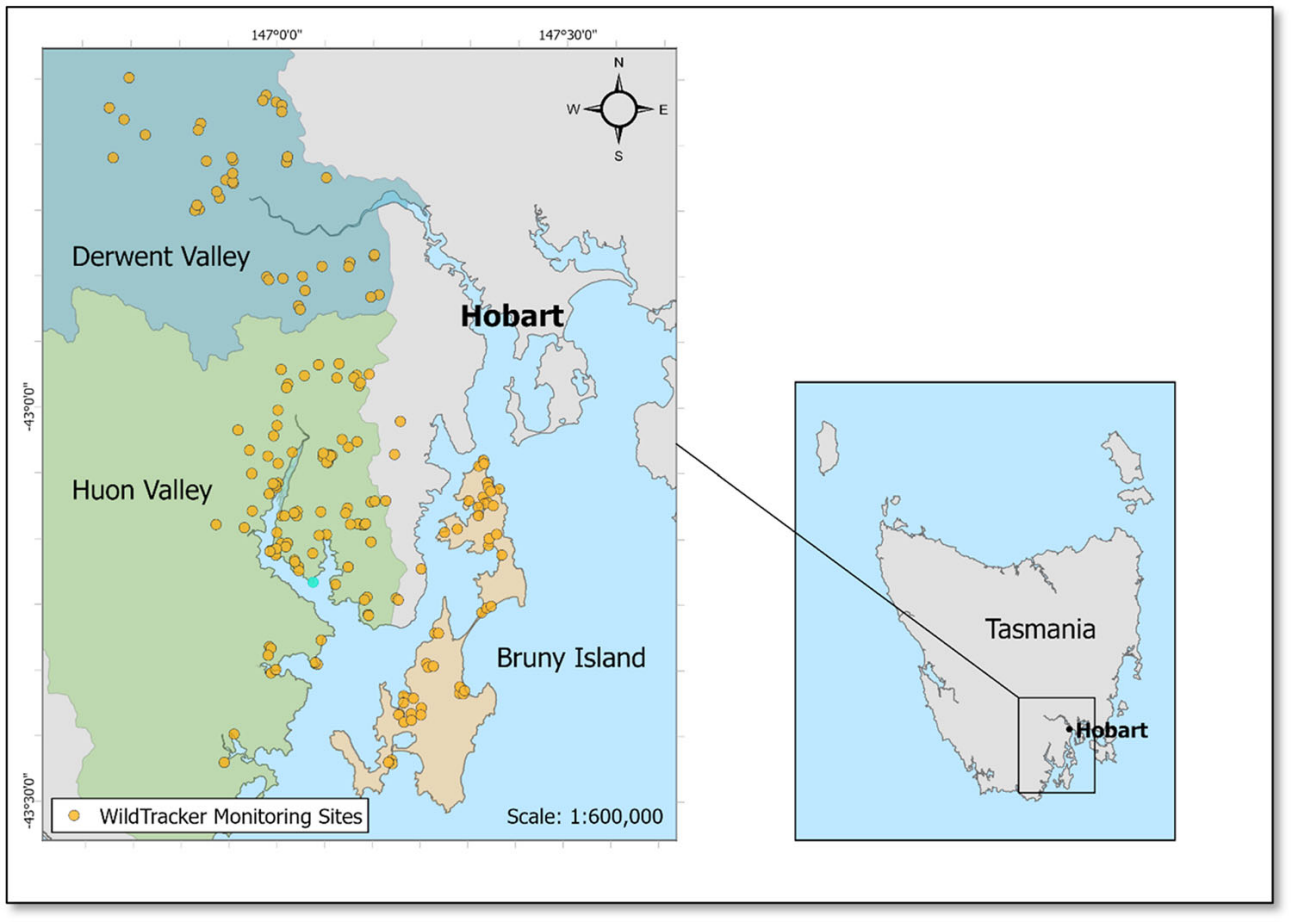
Study regions in southeast Tasmania and approximate location of the properties of participating landholders

The research was undertaken in southeast Tasmania, Australia, an area characterised by rich biodiversity, productive agricultural lands, and dynamic human-nature interactions. Tasmania is a large maritime island off the south coast of Australia, with a cool temperate climate. Tasmania has been continuously occupied by Aboriginal people for at least 40,000 years (Jones et al. 2019). Following European colonisation, agriculture development has transformed much of the Tasmanian landscape. Tasmania has diverse wildlife communities, including species of mammals that remain reasonably common and widespread (with some localised declines) but are rare or extinct in their former ranges on mainland Australia. Large areas of land area under private ownership but with a high retained cover of native (variously modified) vegetation, and potentially high biodiversity value. Regional differences in agricultural practices and the extent of native vegetation clearance have influenced the distribution of native species and habitats. The mosaic of private land allotments, many small in extent, are subject to a wide variety of management styles that create a socio-ecologically heterogeneous landscape. The study focused on three distinct regions: the Huon Valley, Bruny Island, and the Derwent Valley (Fig. 3). These regions were selected in order to compare and contrast the influence of biogeographic and socio-economic characteristics on species distributions and habitat condition.

### Recruitment of Landholder Participants

Private landholders with properties of at least one hectare in size were recruited via the networks of the Tasmanian Land Conservancy and through advertising on community noticeboards, traditional and social media. The project was titled ‘WildTracker’ and has since evolved from the pilot phase reported herein into an ongoing citizen-science program hosted by the Tasmanian Land Conservancy. A total of 160 landholders participated in the research. A concerted effort was made to involve landholders representing a wide cross-section of the Tasmanian rural community, including farmers, lifestyle property owners, long-term residents, and recent arrivals to the study areas. All landholders attended a training workshop, which outlined the research objectives and provided hands-on training in techniques for collecting data on mammals, birds, and habitat, including the use of motion-sensor cameras, sound recorders, and photo-point monitoring. Some landholders also participated in other elements of the research, including through interviews, a problem-framing workshop, socio-economic and land management survey, and classification of wildlife images and habitat photos. Recruitment and participation in the research were in accordance with Human Ethics Permit H0016014, issued by the University of Tasmania.

### Sampling Strategy and Site Selection

Landholders were provided with a property map that showed the distribution of broad vegetation or habitat types. They were instructed to establish a long-term monitoring site in one or more habitat types, depending on the size and ecological characteristics of the property. Sites were located at least 500 m apart to mitigate spatial autocorrelation of the species distribution data. Landholders were provided with instructions on how to choose a suitable site for mammal and bird observations using the supplied equipment but were also encouraged to use knowledge of their property and local wildlife to guide the exact placement of the site. Further details of survey procedures for ecological indicators are provided in Table 1.

**Table 1:** Site indicators, survey method and procedures for collection of ecological data

Site Indicator	Survey method	Procedure
Mammals	Landholder survey – wildlife camera	Camera type: ScoutGuard SG560K Deployment period: 21 days Trigger setting: multi-shot (3 photos) Lapse period: 30 s Lure: Scent attractant Deployment location: on track (vehicle/walking/ animal)
Birds	Landholder survey – sound recorder	Recorder type: Zoom H2N or smartphone Recording period: 20 min Recording time: +\− 60 min of dawn Recoding location: same as for wildlife camera
Habitat condition	Landholder survey – photo monitoring	Camera type: personal digital camera or smartphone Photo orientation: north, south, east, west

### Field Collection of Ecological Data

Ecological data were collected by landholders on mammals, birds, and habitat condition. These site-scale indicators were selected because they are of high conservation significance, were identified as important natural values by landholders, and survey technologies and procedures are available that facilitate a citizen-science approach to both data collection and analysis. Equipment was provided by the Tasmanian Land Conservancy in four rounds, with a limited supply of equipment rotated between landholders over a 16-week period in the Tasmanian spring and summer. Landholders were provided with a field manual containing instructions on field survey techniques, and support could be accessed from the research team via phone or email. Field data were collected in accordance with Animal Ethics Permit A0015788, issued by the University of Tasmania.

### Landscape Indicator Calculation

Spatial analyses were used to characterise the landscape context characteristics of each site, including vegetation cover, land productivity, and water availability. These indicators were identified in the stakeholder workshop as most important for determining distribution and abundance of native wildlife. Publicly available vegetation, agricultural and hydrological datasets were accessed from the Land Information Systems Tasmania database (Department of Natural Resources and Environment Tasmania 2023). Landscape indicators were analysed using geoprocessing functions in ArcGIS Pro software. Details of landscape indicators, source datasets and calculation procedures are detailed in Table 2.

**Table 2:** Landscape indicators, source datasets and calculation procedures

Landscape Indicator	Geospatial source data	Procedure
Native vegetation type (9 categories)	TASVEG 4.0 – Vegetation Group	*Spatial join* geoprocessing function
Native vegetation extent (percentage)	TASVEG 4.0 – Modified Vegetation Category	*Buffer, Clip* and *Calculate Geometry* geoprocessing functions (100 m, 250 m, 500 m, 1 km, 2 km, 5 km)
Riparian distance (metres)	LIST Hydrology LIST Hydrography	*Near* geoprocessing function
Land productivity (7 categories)	Land capability	*Spatial join* geoprocessing function

### Socio-Economic and Land Management Survey

Socio-economic and land management data were collected via a survey sent to the 160 landholders participating in the WildTracker program. The survey questions were based on factors identified as most important during the stakeholder workshop (Table 3). The survey was also sent to 1066 neighbouring landholders within 500 metres of a data collection site, because the workshop identified neighbouring land management as a potentially important driver of wildlife conservation outcomes. In total 454 responses to the survey were received (response rate 57%).

**Table 3:** Landholder survey categories, sub-categories, and question types

Indicator Category	Sub-category	Question type
Property information	Property size Ownership time Proportion of income earned from property Residential status Natural resources Environmental values	Area in hectares or acres Years of ownership 5 categories Resident/absentee 5 categories plus ‘other’ 6 categories plus ‘other
Land management	Property type Primary land manager Land management objectives Land management activities (current) Land management activities (historic) Land management time Land management expenditure Environmental / NRM program participation	5 categories plus ‘other’ 4 categories plus ‘other’ 6 categories plus ‘other’ 15 categories plus ‘other’ 15 categories plus ‘other’ 4 categories plus ‘other’ Dollars per month 6 categories plus ‘other’
Landholder information	Age Gender Country of birth Weekly household income Education Occupation	8 categories Open ended response Open ended response 8 categories 5 categories plus ‘other’ Open ended response
Environmental values	New Ecological Paradigm Scale (Dunlap and Van Liere 1978) Sense of place	15 questions, Likert scale rating Open ended response
Local community	Community organisation participation Common interests Community relationships	9 categories plus ‘other 3 categories 3 categories
Local ecological knowledge	Sources of knowledge Trustworthiness of knowledge sources Types of knowledge	8 categories plus ‘other’ 7 categories, Likert scale rating 12 questions, Likert scale

### Preliminary Ecological Analysis

Landholders collected raw data on mammals, birds, and habitat condition in the form of photos and sound recordings. Mammal and habitat images were classified by landholders and a team of volunteers, hosted by the Tasmanian Land Conservancy. Training sessions were provided by the research team and a guidebook was prepared to aid mammal identifications. Volunteer-classified images were validated by the research team through a hierarchical review process that focused on identifying unclassified images, then checking rare species, before finally checking a subset of commonly recorded species. In total volunteers classified 35,431 fauna detections from 160 camera deployments.

An activity index for each species was calculated for each site as the proportion of days that the species was detected during a camera deployment. Total richness of mammals and feral animals was also calculated for each site and aggregated across deployments for properties with multiple survey sites.

As a cross-validation procedure, the images were also classified using a recently developed deep-learning algorithm that had been trained on a dataset of over one million tagged images from parallel University of Tasmania fauna research projects (Brook et al. 2023). Cross validation of species detected at least 50 times showed a mean discrepancy between classification datasets of 11.7%. Rarer species were proportionally more likely to be misclassified by both human and AI recognisers, highlighting the importance of cross-validation measures in wildlife citizen-science projects.

Sound recordings were reviewed by a skilled volunteer ornithologist and presence, or absence of bird species was recorded for each site. The sound recordings were validated by review of a proportion of recordings by a trained ornithologist. The acoustic dataset yielded 1165 observations from 84 sites.

Habitat photos were reviewed by volunteers and were visually classified according to structural characteristics (density of three vegetation strata), and the proportion of native to introduced plants. classification system is shown in detail in Table 4.

**Table 4:** The classification system used to analyse photo-point images of habitat

Vegetation structure		Nativeness
Strata	Density	
Understorey	Low density <30%	Mostly exotic species ( < 30% native)
Mid-storey	Medium density 30–70%	Mix of species (30–70% native)
Canopy	High density > 70%	Mostly native species ( > 70% native)

### Property Reports and Feedback/Feedforward Workshops

Property reports were compiled by Tasmanian Land Conservancy volunteers and provided to each landholder participating in WildTracker. The reports contained summary and descriptive statistics, such as habitat condition, the number of native species, the number of feral species, the relative abundance of each species, and expected species that were not detected. The expected species list was determined from species distribution maps for Tasmanian fauna (Rounsevell et al. 1991). A property map and management recommendations were also included in each report.

Feedback/feedforward workshops were held in each participating region, in order to present the preliminary findings of the survey and identify future opportunities for improving the processes and areas of focus of the WildTracker program. Summary and descriptive statistics were calculated for each region in order to compare and contrast the findings of the fauna and habitat surveys at a regional scale. There were clear differences in the patterns of distribution and abundance of many species, provoking a discussion about potential causes, both natural and anthropogenic. The preliminary survey findings were also compiled into a report that was circulated to WildTracker participants (Taylor 2017).

### Detailed Analysis of Ecological Components of the Social-Ecological System

Detailed analysis of social-ecological datasets followed an iterative process, guided by the focal relationships identified as most important by the stakeholder-developed social-ecological system conceptual model (Table 5). Analysis focused on the ecological and land management components of our social-ecological systems model. Social factors were considered as secondary drivers that effect primary drivers like vegetation cover and fragmentation. Calculations were done using the R statistical package (R Core Team 2023).

**Table 5:** Description of analytical procedures for detailed social-ecological analysis

Analysis	Description
Correlation plots	To assess relationships, correlation plots were used to identify linear correlations in ecological data. We used the Pearson correlation coefficient to measure continuous dependent variables (e.g., species richness, species activity index) and independent variables (e.g., cat abundance, landscape characteristics, landowner survey data). For analysing relationships between continuous dependent variables and binomial categorical independent variables (e.g., region, land cover type, management practices), we used the point-biserial correlation method.
Non-metric multidimensional scaling (NMDS)	For cluster analysis, we did Non-Metric Multidimensional Scaling (NMDS) analysis using the metaMDS function from the *vegan* package in R. NMDS is a distance-based ordination method that transforms the data matrix into a dissimilarity matrix, forming the basis for ordination. For this analysis, dissimilarity matrices were calculated for native mammal and bird activity, with rows representing sites and columns representing species. The NMDS procedure is iterative, starting with defining original positions in multidimensional space and creating an initial configuration in reduced dimensions (usually 2D). The initial configuration is refined through regression against observed distances, with stress as a measure of disagreement. Stress levels guide the quality of dimensionality reduction, with lower values indicating better representation. Final NMDS plots were generated for both groups, with points coloured by various landscape, site, and landowner survey variables to identify potential groupings.
Random Forest machine learning	For prediction, we used Random Forests machine learning via the *randomForest* function from the *caret* package in R (Kuhn 2008). Random Forests, an ensemble of decision trees, employs recursive partitioning on subsets of training data and features. This approach introduces randomness, enhancing diversity and mitigating overfitting. In determining appropriate data splits, the trees contribute to an average prediction, generally yielding more accurate and robust results than individual decision trees. The analysis focused on identifying important variables in datasets including native mammal richness, native bird richness, and the abundance of both rare (eastern quoll, eastern bettong, eastern barred bandicoot, long nosed potoroo) and common species (Bennetts wallaby, bare-nosed wombat, grey currawong). For each independent tree, the dataset was divided into training (70%) and testing (30%) sets. Model tuning was via the *tuneRF* function to optimize the number of trees (*ntrees*) and the number of variables tried at each split (*mtry*). The fit of the models was assessed using variance explained, and variable importance plots along with partial dependency plots to examine the relationships between predictor (landscape, site, and landowner variables) and dependent variables (species richness or abundance). Given the limited number of sites contributing landowner variables, these were analysed separately from other landscape and site variables, ensuring a focused and relevant examination of their impact.

## Results

The integration of diverse datasets including landholder-contributed data presented analytical challenges. Under the WildTracker participatory citizen-science model, the research collated extensive data from 160 properties and 285 sites. This approach, while robust in data collection, encountered gaps and inconsistencies across sites, a common issue in social-ecological and citizen science research (Dickinson et al. 2012; Guerrero et al. 2015). In this study, these issues were manifested in a high number of moderately significant predictor variables. This emphasised the need for more uniform data collection methods and highlighted the intricacies of balancing the depth and breadth of data in complex systems. Overcoming these difficulties are crucial to understanding the dynamics affecting mammal and bird assemblages in privately managed landscapes, and they underscore the trade-offs inherent in such comprehensive social-ecological research endeavours.

### Summary and Descriptive Statistics

**Fig. 4:**
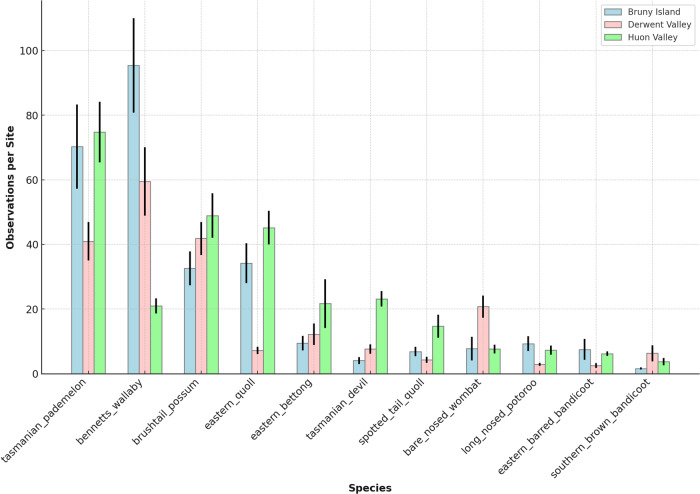
Frequency of detection per region of eleven target native marsupial species, ranked from most frequently detected species to least frequently detected species

**Fig. 5:**
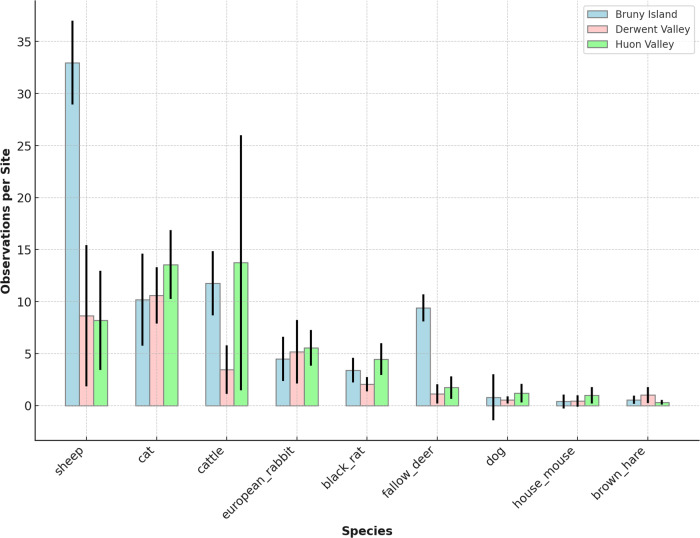
Frequency of detection per region of introduced mammal species, ranked from most frequently detected species to least frequently detected species

A total of 52 species were identified by the wildlife camera survey. This included 40 native species and 12 introduced species. Of the native species, 18 were mammals and 22 were birds. Of the introduced species, 9 were mammals and 3 were birds. Significant regional differences in the relative activity of native mammals (Fig. 4) and introduced mammals (Fig. 5). The most common native mammal species are generalist herbivores such as the Tasmanian pademelon (*Thylogale billardierii*), Bennetts wallaby (*Macropus rufogriseus*) and brushtail possum (*Trichosurus vulpecula*), which were frequently detected across all study regions. Threatened species such as the eastern quoll (*Dasyurus viverinus*), Tasmanian devil (*Sarcophilus harrisii*) and eastern barred bandicoot (*Perameles gunnii*) were less frequently detected. The Huon valley remains a relative stronghold for these species. Some species listed as least concern, such as the bare-nosed wombat (*Vombatus ursinus*), long-nosed potoroo (*Potorous tridactylus*) and southern brown bandicoot (*Isoodon obesulus*) were among the least frequently detected species. Note that Bruny Island has a naturally lower diversity of native mammal species: species such as the Tasmanian devil and spotted tailed quoll were absent from the island at the time of European colonisation.

Data on both domestic and feral animals are presented here in order to show the relative abundance of these species in comparison to native fauna. Sheep and cattle are the most abundant domestic livestock species in southeast Tasmania. Sheep were most frequently detected on Bruny Island where there are extensive grazing properties on the northern part of the island. Cats were second most frequently detected introduced species, despite their typically low density and cryptic nature. They are detected at a similar frequency across all three study regions. Fallow deer were detected most frequently on Bruny Island and is noteworthy in that they are a recently established population following a documented escape into the wild.

**Fig. 6:**
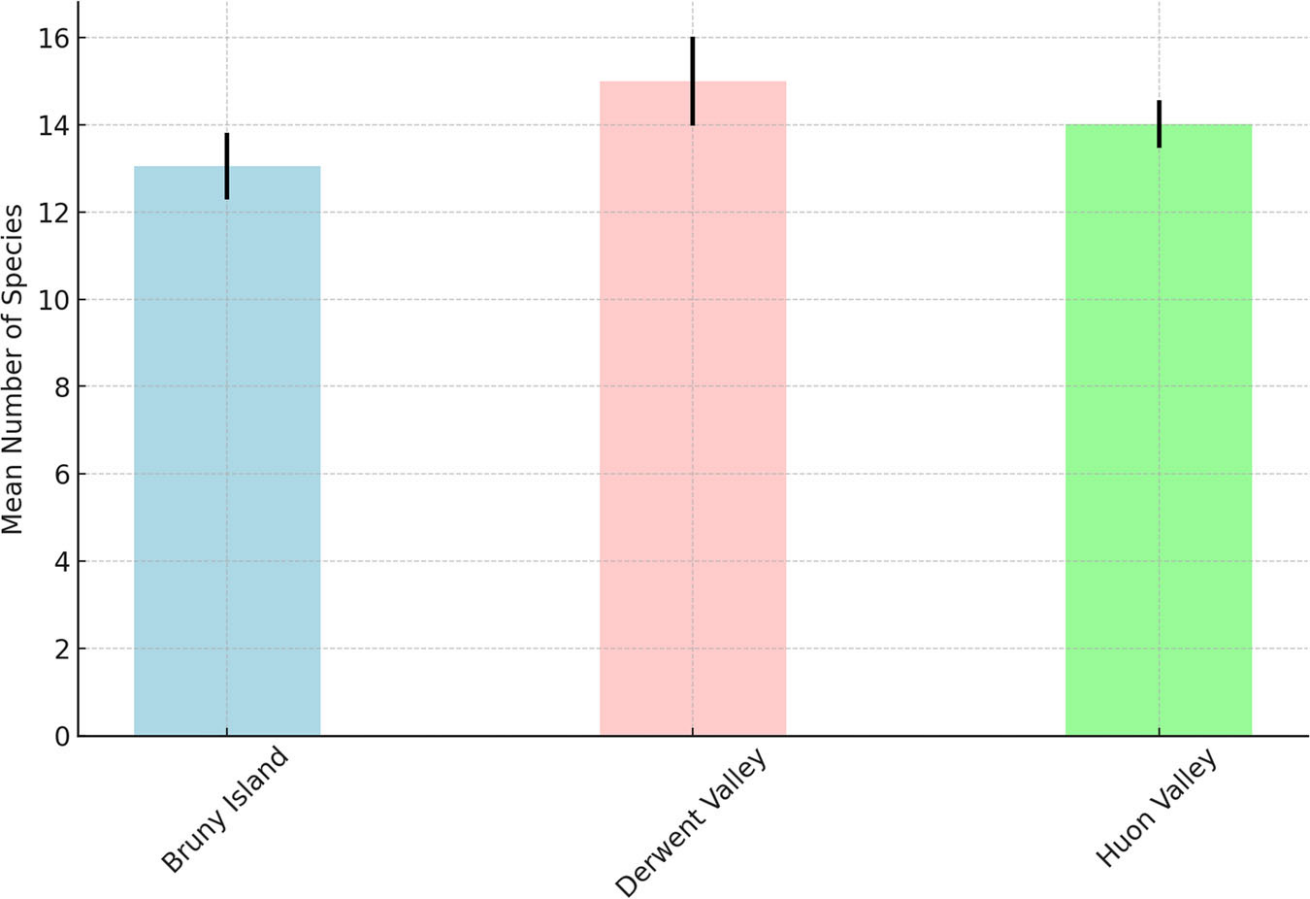
Mean number of bird species per site by region with Standard Errors. This bar chart shows the average number of bird species observed per site in each region. Error bars indicate the standard error of the mean, providing a measure of the variability in the data across different sites within each region

**Fig. 7:**
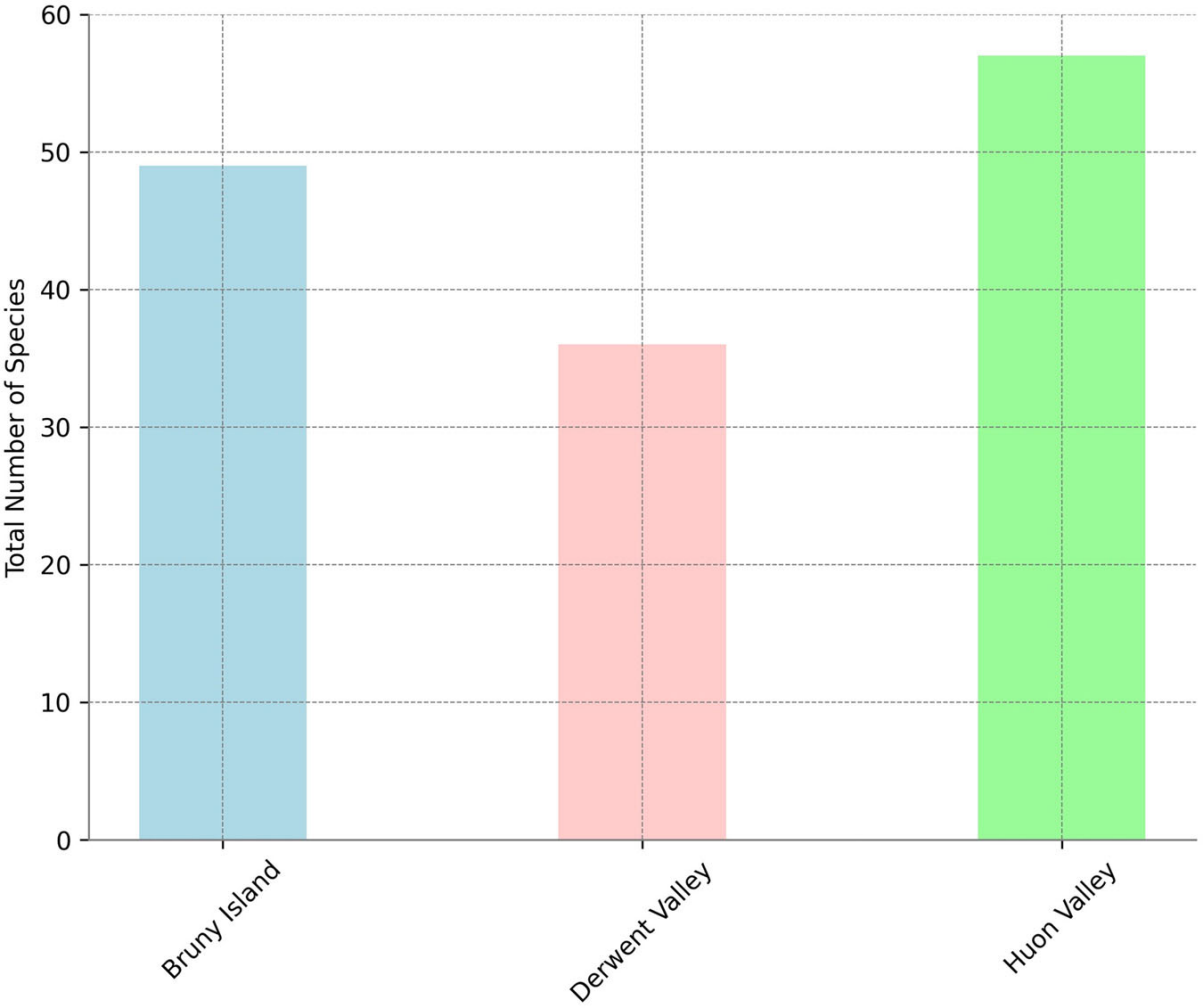
Total number of bird species recorded by each region. The bars represent the cumulative count of distinct species observed

A total of 64 species of birds were identified by the acoustic survey. This included 54 native species, seven of which were Tasmanian endemic species, and ten introduced species. Three threatened species were identified: the swift parrot *(Lathamus discolor)* was identified at 15 sites, the blue-winged parrot (*Neophema chrysostoma*) was detected at five sites, and grey goshawk (*Accipiter novaehollandiae*) was identified at one site. The most frequently detected species was the forest raven (*Corvus tasmanicus*), which was detected at 92% of sites. The ten most frequently detected species were all native and were detected at >50% of sites. The most frequently detected introduced species was the common blackbird (*Turdus merula*), which was detected at 49% of sites. Three introduced species of management concern were detected: the rainbow lorikeet, superb lyrebird, and Eurasian starling. The rainbow lorikeet and superb lyrebird are native to the Australian mainland but were introduced to Tasmania. These species potentially impact native species via competition for nesting and foraging resources, and habitat alteration (lyrebird). Rainbow lorikeet and Eurasian starling Species richness per site was greatest in the Derwent Valley (Fig. 6), but the Huon Vally recorded the highest diversity of bird species (Fig. 7).

### Socio-Ecological Drivers of Wildlife Conservation – Land Management and Ecological Components

**Fig. 8:**
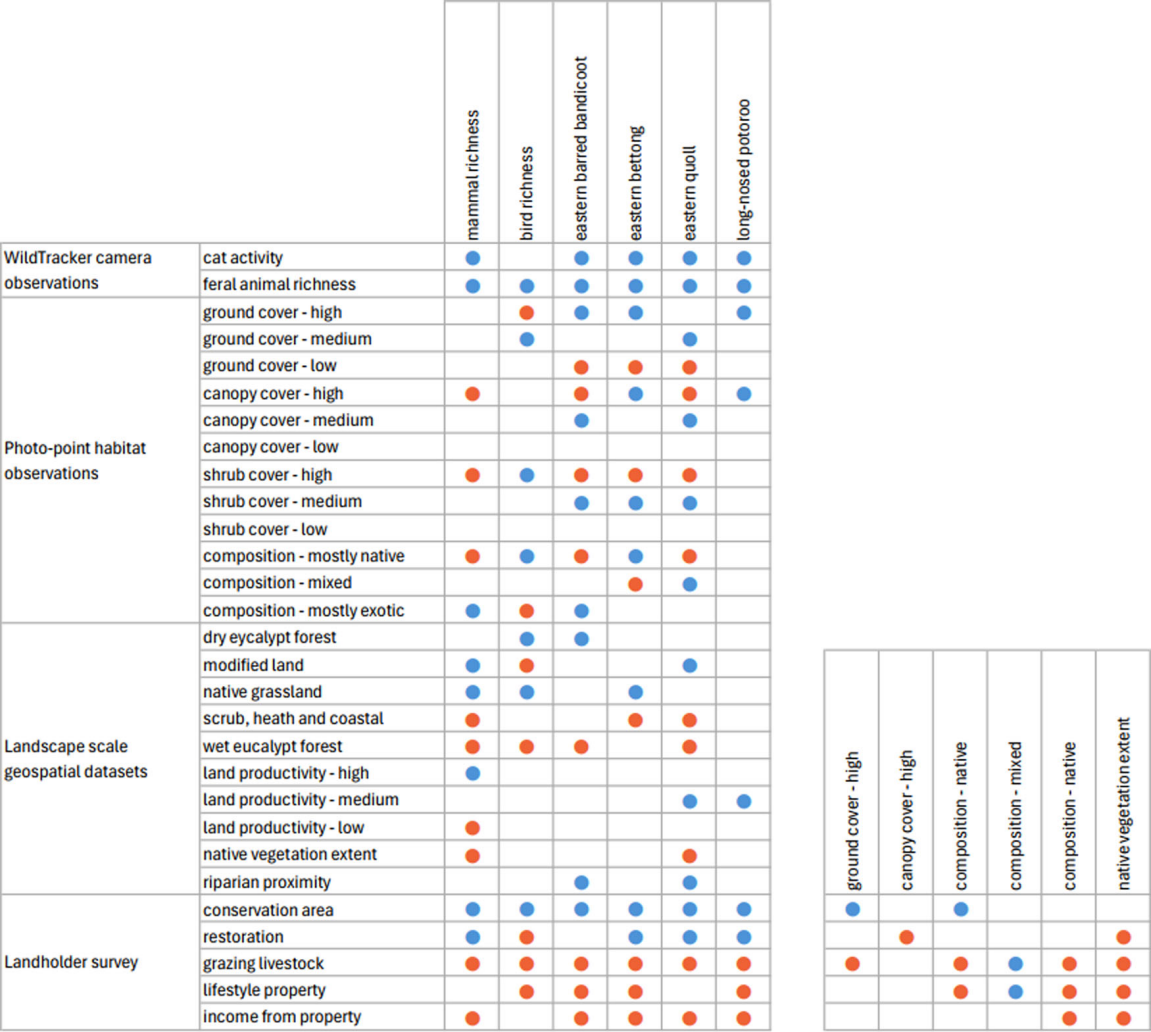
Summary of correlation plot results for mammal richness, bird richness, focal species against a subset of predictor variables for which a moderate (0.2) to strong (1.0) correlation was observed. Correlations between land management and vegetation predictors are also shown. Blue marks indicate a positive correlation, and red marks indicate a negative correlation

Correlation plots of mammal and bird richness with site and landscape variables showed low to moderate correlations across a wide range of variables. The focal species showed intercorrelation amongst those species, and stronger correlations between them and site factors than landscape factors. Common species (Bennetts wallaby, common wombat, brushtail possum, grey currawong) all showed week relationships with predictor variables, indicating their ubiquity in the landscape (low habitat selectivity). Correlations were also observed between land management predictors and site and landscape scale vegetation predictors. These variables are the nexus between ecological and social components of the social-ecological system. Correlations between social-ecological predictor variables and mammal richness, bird richness, and focal species activity index are shown in Fig. 8.

**Fig. 9:**
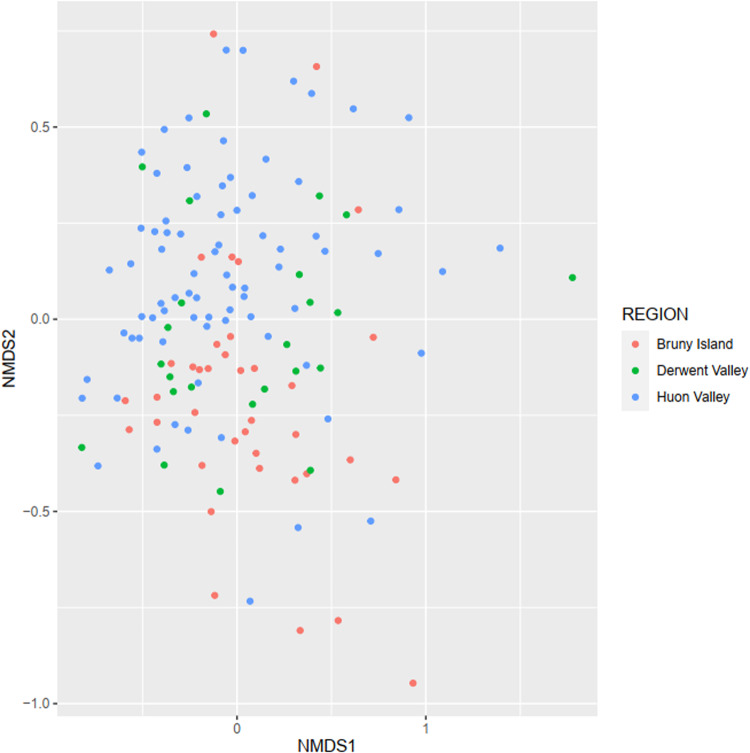
NMDS plot of native mammals with sites colour coded by region. Bruny Island shows a moderate differentiation from the other two study regions, although many of its sites were not separable

**Fig. 10:**
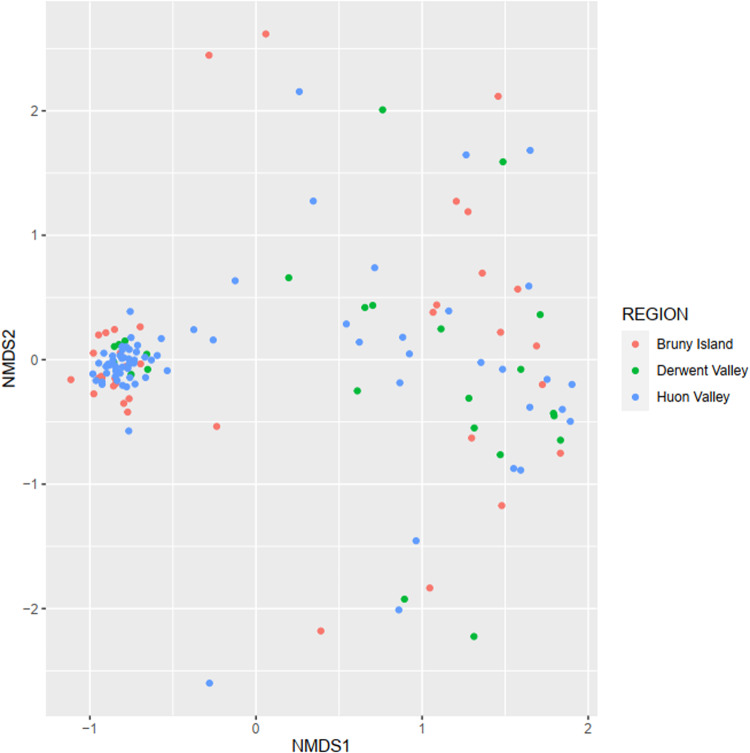
NMDS plot of native birds with sites colour coded by region. Regions show no obvious differentiation

Non-metric multidimensional scaling of mammal and bird observation data showed no obvious differentiation of fauna into distinctive communities across the study area when visualised against the majority of predictor variables. The only clear grouping separated the Bruny Island mammal assemblage from the other study regions (Fig. 9), which is to be expected given that it is an island with a naturally depauperate fauna. This community separation was not observed for birds, which are able to transit the narrow channel between the island and mainland Tasmania (Fig. 10).

**Fig. 11:**
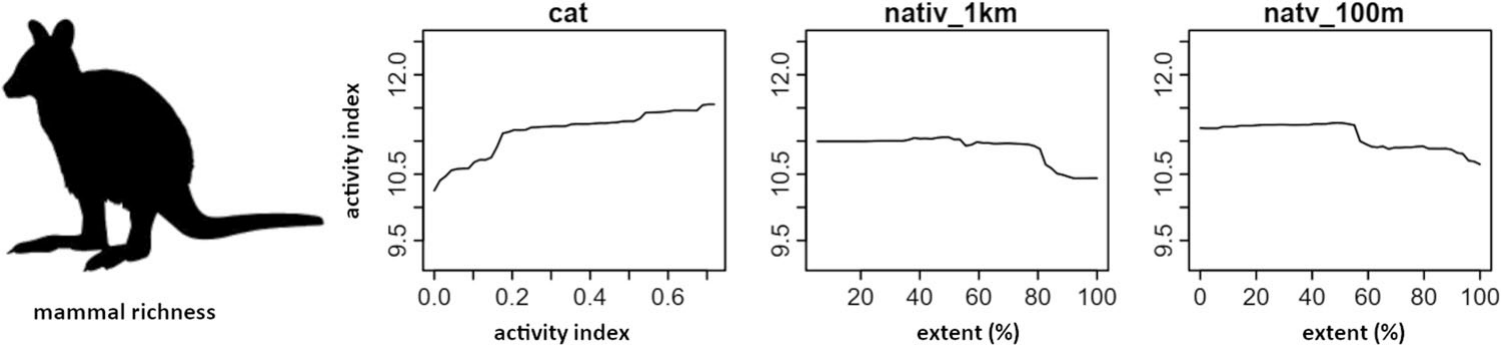
Mammal richness dependency plots for the three most significant social-ecological predictor variables from RF analyses: cat activity (cat), native vegetation extent within 1 km of a site (native_1 km), and native vegetation extent within 100 m of a site (natv_100m)

**Fig. 12:**
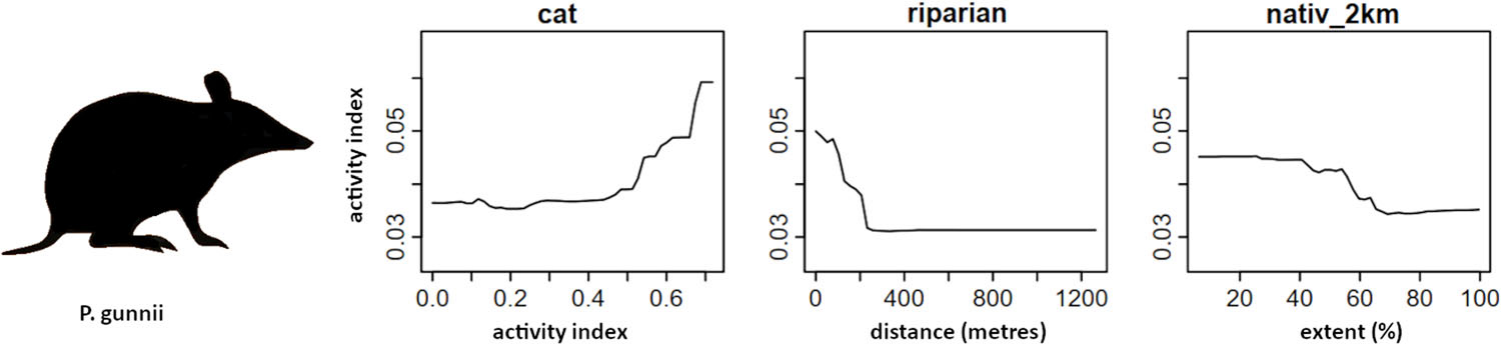
Eastern barred bandicoot dependency plots for the three most significant social-ecological predictor variable from RF analyses: cat activity (cat), distance from stream or waterway (riparian), and native vegetation extent within 2 km of a site (nativ_2km)

**Fig. 13:**
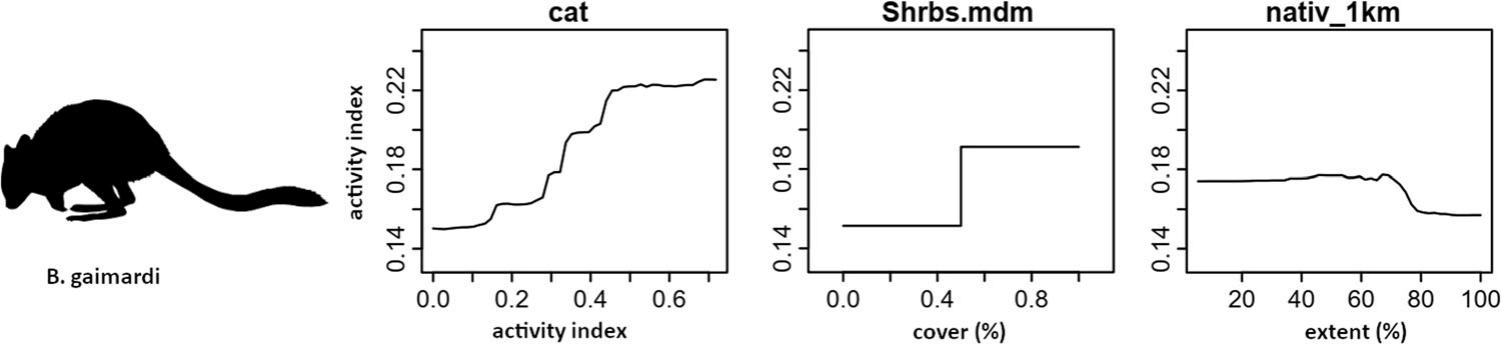
Eastern bettong dependency plots for the three most significant social-ecological predictor variable from RF analyses: cat activity (cat), shrub cover (Shrbs.mdm) and native vegetation extent within a 1 km of a site (nativ_1 km)

**Fig. 14:**
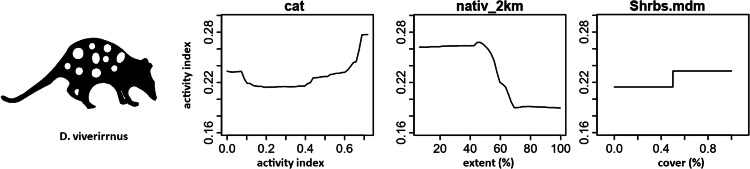
Eastern quoll dependency plots for the three most significant social-ecological predictor variable from RF analyses: cat activity (cat), native vegetation extent within 2 km of a site (nativ_2 km), and shrub density (Shrbs.mdm)

**Fig. 15:**
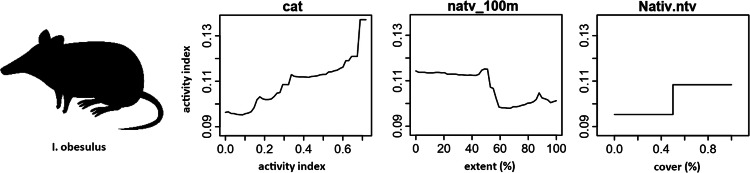
Long-nosed potoroo dependency plots for the three most significant social-ecological predictor variable from RF analyses: cat activity (cat), native vegetation extent within 100 m of a site (natv_100 m), and proportion of native vegetation at a site (Nativ.ntv)

Random forest (RF) analysis confirmed the importance of relationships between predictors and dependent variables identified through correlation plots. Dependency plots show non-linear relationships and evidence of ecological thresholds, especially for the native-vegetation extent landscape variables. The activity level of cats was found to predict both mammal richness and the activity of focal mammal species. The same relationship was also found for introduced animal richness. RF analysis also identified additional predictor variables of importance for native mammals relating to the extent of native vegetation within a radius of a monitoring site. Mammal richness declined when native vegetation cover within 1 km of a site decreased beyond 80%, and when native vegetation within 100 m of a site decreased beyond 50% (Fig. 11). Eastern barred bandicoot activity decreased sharply with distance from a stream or waterbody, and when native vegetation within 2 km of a site decreased below 50% (Fig. 12). Eastern bettong activity increased with increasing shrub cover, and decreased when native vegetation within 1 km of a site was below 70% (Fig. 13). Eastern quoll activity decreased substantially when native vegetation extent within 1 km of a site was below 50% and increased with increasing shrub cover (Fig. 14). Long-nosed potoroo activity decreased when native vegetation within 100 m of a site decreased below 50% and increased within an increasing proportion of native understorey vegetation (Fig. 15). The importance of predictor variables is covered comprehensively in the Supplementary Materials.

## Discussion

The principal objective of the WildTracker research collaboration was to identify key social-ecological drivers influencing wildlife conservation populations on private land. This was achieved through a participatory research methodology, which actively involved stakeholders in co-design, data collection and analysis. A co-designed conceptual model of the social-ecological system (Fig. 2) provided interrelated hypotheses which were tested through an iterative analytical process. We identified several distinct and significant relationships within elements of the conceptual model and this research advances understanding of both the drivers of wildlife conservation on private lands and the practices of collaborative research.

Our research highlights the importance of ‘messy,’ ecologically heterogeneous, human-dominated landscapes for wildlife conservation. Native wildlife can tolerate or thrive in the highly modified habitats that have been created by people in rural landscapes. The embedded nature of humans in social-ecological systems can’t be ignored, and is reflected in an interview statement from one of the participating landholders:

“This property taught me a lot about a relationship with nature… There is absolutely no distinction between managed and natural areas… This landscape is in fact highly humanized, and it has been for thousands of years”

The following discussion is structured according to our guiding conceptual model. It focuses on major findings on the relationships between land management, ecological pressures at the landscape and site scale, and wildlife outcomes. These elements of our model showed the most significant relationships. While important, the relationship between socio-cultural attributes of landholders were less clearly established by our analyses and warrant further investigation. Additionally, the important role of citizen scientists in wildlife monitoring and management is discussed, and we advocate for a greater role for private landholders in landscape scale wildlife monitoring and management.

### Land Management Practice: A Nexus within Social-Ecological Systems

Land management include a suite of socio-economic and cultural practices. This element of our social-ecological model serves as a critical nexus in the interaction among humans, habitats, and wildlife. Human activities have profoundly modified private landscapes creating ecologically heterogeneous environments. Conventional conservation theory would indicate that the impact of this modification on native wildlife would be deleterious. However, we found a mix of positive and negative associations with various categories of land use. Utilising survey data from landholders, our study investigated the dynamics of land management and its implications for wildlife conservation. We observed a complex impact of land management on wildlife populations, revealing interactions among property size, land use, and wildlife dynamics. Notably, not all examined factors were important to wildlife, and the relationships presented here are relatively weak. Our analysis categorises land management factors into primary, active factors like grazing, invasive species management, and restoration, and secondary factors including property type, time spent on land management, property size, and income from land.

The most significant predictor of wildlife outcomes related to grazing, which correlated with negative outcomes for all fauna indicators. Grazing correlated with larger properties and higher farm income. It impacts fauna populations by degrading understorey habitat quality and extent (Kirkpatrick et al. 2005) and is linked to broader landscape scale drivers such as native vegetation clearance and fragmentation (Eichenwald et al. 2020). Grazing management was associated with larger properties and greater farm income. Note that our study did not quantify grazing intensity, and research suggests that some grazing strategies are conducive to wildlife and habitat conservation (Leonard and Kirkpatrick 2004). In contrast, properties dedicated to conservation purposes, while associated with less time spent on land management, exhibited higher diversity of mammals and birds, and were positively correlated with our four focal mammal species. This emphasises the vital role of private conservation lands in complementing and connecting public reserve systems and nature conservation initiatives (Ivanova and Cook 2020; Bingham et al. 2021). Interestingly, bird diversity was negatively correlated with the presence of active or historic native vegetation restoration on a property, an effect not observed in mammals. Restoration was negatively correlated with vegetation extent and site-scale habitat condition variables. Revegetation typically occurs in highly cleared and fragmented landscapes (Davidson et al. 2021), and there has been a historic tendency in restoration initiatives to plant primarily canopy tree species rather than diverse understorey that provides structural complexity (Lindenmayer et al. 2018; Jones et al. 2021). This can favour aggressive forest and woodland birds if there is an absence of suitable cover for smaller species such as honeyeaters (Munro et al. 2007; Bennett et al. 2022).

The variation in responses between bird and mammal species underscores the need for targeted conservation strategies that address the unique needs of different faunal groups. The historical context of land management is also critical, as past practices may continue to influence present ecological conditions (Race et al. 2012). The participation of landholders in the research process as survey respondents and data collectors provides valuable insights but may introduce a self-selection bias, a factor that must be considered when interpreting these results (Pateman et al. 2021). Landholders with a disinterested or antagonistic attitude to wildlife are unlikely to have engaged in our conservation-centric project. This study highlights the diverse and sometimes counterintuitive effects of land management practices on wildlife conservation. The diversity of management approaches evident in private landscapes determines landscape-scale patterns in the extent, configuration, and condition of habitats for wildlife and is therefore a fundamental social-ecological driver of wildlife conservation outcomes.

### Landscape-Scale Ecological Pressures: Thresholds of Habitat Loss and Fragmentation

Agricultural and residential development have profoundly modified native ecosystems at landscapes scales (Mackey et al. 2013; Magioli et al. 2016). The most damaging impact has been the conversion and degradation of habitat (Legge et al. 2023). Despite these impacts, this study shows that wildlife is resilient and can persist in landscapes that have been significantly modified by human activities. A key finding of this research is the distinction between the ecological pressures when viewed at the site versus landscape scales. While site-specific factors influence immediate habitats, landscape-scale considerations are pivotal in shaping broader ecological networks and corridors. These larger-scale factors significantly affect faunal movement, genetic diversity, and long-term species viability, emphasising the need for a strategic landscape-scale approach to wildlife conservation (Downes et al. 1997; Mackey et al. 2013; Davidson et al. 2021).

One of the principal landscape-scale factors influencing mammal and bird diversity is the intactness of native vegetation within a radius of a site. Our findings confirm that both mammal and bird assemblages can tolerate a relatively high degree of fragmentation (Fischer and Lindenmayer 2006), with this tolerance extending from hundreds of meters to kilometres from a detected location. However, there are critical thresholds for native vegetation loss, beyond which species richness at the landscape level diminishes (Saunders et al. 1991; Fischer and Lindenmayer 2007). We found that at certain degree of native vegetation loss, a decline in species richness at a site becomes evident. This pattern was observed for the focal mammal species, with the distance and the percentage threshold of intact habitat varying between species. For instance, the activity of the long-nosed potoroo showed a sharp decline when native vegetation within 100 meters of a site dropped below 50%. This aligns with other studies indicating the species’ preference for intact forest areas (Norton et al. 2010).

Conversely, the activity of eastern quolls and eastern barred bettongs declined significantly at sites where there was a loss of more than 50% of native vegetation within a 2 km radius. Species such as the eastern quoll and eastern barred bandicoot are more tolerant of landscape scale disturbance compared to the long-nosed potoroo. The contrast in conservation status, with both the quoll and bandicoot being threatened while the potoroo is not (although it is patchily distributed), underscores the importance of managing site-scale factors in conjunction with landscape-scale vegetation configuration. This finding supports findings from quantitative and expert elicitation analyses of dispersion in Australian species (Jones and Davidson 2016; Lechner et al. 2017). Such an understanding of species-specific thresholds can inform evidence-based landscape scale conservation planning, tailoring strategies to the unique ecological needs of each species (Noss 2008; Lechner et al. 2017; Proft et al. 2018; Gardiner et al. 2019) and thereby helping wildlife to persist in modified and heterogeneous private landscapes.

### Site-Scale Ecological Pressures: The Importance of Productive, Mixed-use Lands

Site scale habitat condition is a significant ecological pressure on wildlife populations. Our study found that many wildlife species are able to persist and even thrive in highly modified habitats at the site scale. Some groups and taxa even displayed a preference for modified habitats, highlighting the importance of productive landscapes where mixed agricultural and residential land uses dominate. Mammals were more diverse in areas of high land productivity, regardless of the composition of the vegetation, and were positively correlated with the *Modified Land* vegetation category. The eastern quoll and eastern barred bandicoot (both threatened species) showed a preference for modified land and valley locations in proximity to streams or waterbodies. At face value this finding contradicts an established literature that consistently demonstrates that conversion of habitat leads to decline in native wildlife populations (Johnson et al. 2017; Almond et al. 2022; Legge et al. 2023). Productive landscapes are the focus of agricultural development and human settlement, which has resulted in the loss of a significant proportion of native vegetation.

However, there is a growing literature that recognises the values of mixed agricultural and peri-urban landscapes for faunal conservation (Burel et al. 1998; Dotta and Verdade 2011; Ehlers Smith et al. 2018; Semenchuk et al. 2022). Productive landscapes provide the highest and the most consistent supply of natural resources and historically supported the richest native fauna communities prior to extensive agricultural activities. Many native faunal species are not dependent on native plants for food and are able to coexist alongside people and agriculture, as long as the basic requirements of foraging resources and sheltering habitat are met (Burel et al. 1998; Rodewald 2003; Dertien and Baldwin 2022). Furthermore, introduced plants have been found to provide important habitat (e.g., food, shelter, cover from predation) in the absence of native alternatives (Marris 2013; Ranyard et al. 2018). This finding supports more nuanced approach to conservation that decouples the conservation of fauna from the conservation of native habitats, in favour of a focus on managing specific pressures that threaten native fauna in human landscape on private land.

Contrasting the pattern observed in mammals, two of our focal species, the eastern bettong, and the long-nosed potoroo, exhibited distinct preferences for native habitat. The eastern bettong favoured intact native vegetation with substantial ground cover, while the long-nosed potoroo’s site-scale habitat preferences were less specific, though it did show a preference for areas with ground cover and a closed canopy. Avian diversity also showed a stronger correlation with mixed and undisturbed remnant vegetation, being highest in sites with intact native understorey, and negatively correlated with sites with primarily introduced vegetation. Bird assemblages were more diverse in locations with a higher density of native shrubs. Diverse native habitats provide a greater variety of foraging niches and shelter from larger aggressive birds such as forest raven and noisy minor (Catterall et al. 1997; Lindenmayer et al. 2018; Bennett et al. 2022; Hingee et al. 2022), and our research confirms pervious research on the importance of native understorey habitat for these species. These findings underscore the necessity of a multifaceted approach to fauna conservation and management, one that is attuned to the diverse needs of local species. Effective management hinges on robust monitoring data; without knowledge of the species present on a property or in a specific area, it becomes challenging to devise land management strategies that cater to all species. A ‘diversified strategy’ in conservation management is likely to yield the most resilient outcomes.

### Site-Scale Ecological Pressures: Feral Cats

Invasive species are a significant ecological pressure on wildlife populations. The feral cat is a mid-sized invasive predator that is widespread in Australia. Cats prey on a wide range of taxa, from small rodents, reptiles, and amphibians to small and mid-sized marsupials (Doherty et al. 2017). They are recognised as significant invasive species both globally and especially on offshore islands (Medina et al. 2011; Dickman et al. 2019; Legge et al. 2020). A notable finding of our study was that feral cats were more prevalent in modified landscapes, in areas of higher land productivity and sites with the greatest diversity and activity of native mammals and birds. This aligns with findings from other researchers, such as Hamer et al (2021) who observed that both cats and the native spotted-tailed quoll were abundant in high-productivity areas, which support plentiful prey populations. Notably, mammal and bird richness, as well as the activity of all four focal mammal species, showed positive correlations with high cat activity in our study. This finding seems counterintuitive, because numerous studies have documented the adverse impact of cats on fauna, including their capacity to cause local extinctions and their role in the extinction of many of Australia’s 34 mammal species since European colonisation (Legge et al. 2020).

However, our study also found that the activity of the four focal species was positively correlated with the presence of medium to high-density ground layer vegetation, while being negatively correlated with areas lacking dense ground vegetation, and that bird diversity was similarly linked to the presence of an intact native shrub layer and medium-density ground vegetation. These findings underscore the significance of sheltering habitats in protecting ‘critical weight range’ mammals and other native fauna from predation, supporting the emerging perspective that complex understory habitats enable small to mid-sized Australian mammals to coexist with high densities of feral cats (Cunningham et al. 2019; Radford et al. 2021). Given the prohibitive cost and logistical challenges associated with feral cat control, managing land to maintain understory vegetation emerges as a pragmatic and implementable strategy for conserving native wildlife in Australia and beyond (Lazenby et al. 2021). In modified Tasmanian private landscapes, exotic plants species such as gorse (*Ulex europaeus*) may have an important ecological role to play in wildlife conservation (Ranyard et al. 2018), further supporting our key finding that ‘messy’ heterogeneous landscapes and properties are important for many wildlife species.

### A Role for Citizen Scientists in Wildlife Monitoring and Threatened Species Assessment?

The WildTracker research collaboration was primarily aimed at enhancing the capacity of landholders in wildlife management on their properties, fostering a network that includes landholders, researchers, and conservation practitioners. This approach not only aimed at co-designing locally relevant data gathering tools but also at enabling landholders to address specific, meaningful questions related to local wildlife management issues. The sizeable number of observations of threatened species by WildTracker participants, corresponded to strong community interest in those species, and demonstrates the potential benefits of utilising citizen science in both social-ecological research and wildlife monitoring.

Despite presenting significant logistical, training and data-integrity challenges, the unrealised potential of citizen science in biodiversity research, particularly in filling the data gaps that hinder effective conservation efforts has been emphasised by a substantive literature (Locke et al. 2019; Dissanayake et al. 2019; Fischer et al. 2021). Our findings corroborate this, showing that citizen scientists were adept at identifying both threatened mammal and bird species across various locations, contributing essential data that might otherwise be unavailable. A total of four threatened mammals and three threatened bird species were identified across numerous locations in all three regions. The data presented in this paper is from one year of data, but ongoing participation in WildTracker is now starting to yield information on trends in wildlife populations. This approach, especially in regions like Tasmania where ecological monitoring is under-resourced, presents a promising avenue for enhancing biodiversity tracking. Many jurisdictions, including Tasmania, suffer from a lack of investment in ecological monitoring. The need for additional resourcing of monitoring is also highlighted by the finding that many species categorised as least concern were found in far lower frequency than some endangered species.

There is a case for assessment of least-concern species such as the southern brown bandicoot, a species considered common and widespread, but which was only identified at a small proportion of sites by this study. Although potentially still locally common on intact public lands, our data suggests a significant decline on private lands. The listing of the eastern quoll as endangered further illustrates this point: this iconic species jumped from least-concern to endangered, only because of a targeted research project that documented a significant decline of the species (Fancourt et al. 2013; Fancourt 2016). This underscores the importance of continuous and comprehensive monitoring strategies, a task where citizen science can play a pivotal role (Mckinley et al. 2017). Lack of data is a serious impediment to effective conservation because the prioritisation of environmental investments and policy are often based on the listing status of species. The role of citizen scientists in identifying the range and trajectory of threatened and more common species can help fill this gap, especially in private landscapes of which they are the custodians.

## Conclusion

Our study demonstrates the importance of private lands for wildlife conservation, particularly productive environments where there a mix of land uses including agriculture and human settlement. These landscapes are characterised by their ‘messiness,’ ecologically heterogeneous at both the site and landscape scale. The diversity of land management across these areas creates a complex mosaic that supports a high diversity of wildlife and offers more stable resources such as food and water. This underscores the need for a new model that recognises the value of modified landscapes in wildlife conservation, akin to the concept of ‘rambunctious gardens’ (Marris 2013), where ecologically varied agricultural and peri-urban areas serve as sanctuaries for both people and wildlife. Our research shows how the interaction between ecological, socio-economic, and land management factors shape wildlife conservation on private landscapes in Tasmania and has relevance to global wildlife conservation efforts. Understanding these dynamics is crucial for developing effective conservation strategies that encompass all aspects of socio-ecological systems, including mammals, birds, their habitats, and private landholders’ values and practices (Hull et al. 2023). Innovative solutions that acknowledge the importance of modified and novel ecosystems are critical to reversing the decline of native wildlife populations on private lands.

Participatory initiatives such as WildTracker can play a significant role, empowering local communities with ecological data, knowledge, and management tools. By integrating insights from WildTracker workshops and interviews, we generated specific hypotheses, validated through analytical methods. This blended empirical data with local observations, enhancing our understanding of socio-ecological dynamics. It identified relationships between fauna assemblages and land management practices at both site and landscape scales, emphasising the need to consider local and broader ecological processes in conservation strategies. The significance of spatial scale in wildlife conservation on private lands cannot be overstated. While individual property-level habitat management is important, it often falls short for wide-ranging species that require broader landscape-level conservation actions (Mackey et al. 2013; Lindenmayer et al. 2016). Consequently, successful conservation strategies on private lands require a synergistic approach: one that not only caters to specific local habitat requirements but also promotes collaborative initiatives at the landscape level among property owners.

The accelerating decline of global wildlife populations in the Anthropocene, highlights the critical importance of translating research findings into management practice. The personal nature of that challenge was eloquently voiced by one of our participating landholders:

“That is when you really test your beliefs, when you suddenly start finding yourself fighting, against what you would like to have, and what is really good for the ecosystem.”

While the results of this study contribute to the global academic discourse on wildlife management, an essential stage in our research journey was sharing results with participants. Results were shared via a project report, discussed at a series of workshops, and each landholder received a property-specific report including tailored management recommendations, which have catalysed and enabled tangible conservation action. This closed the loop on our collaborative process of problem framing and social-ecological inquiry. Employing a socio-ecological methodology, which integrates diverse disciplinary perspectives and stakeholder insights, ensures that conservation actions are informed by evidence that is both contextually appropriate at the local level and practical to implement, thereby addressing the varied requirements of wildlife species across private landscapes.

### Supplementary Information

Supplementary Materials

## Data Availability

De-identified interview data, survey data and quantitative ecological data may be made available upon request.
