# Supplementary material for: Wildlife Conservation on Private Land: A Social-Ecological Systems Study
Source: Environ Manage. 2024 Mar 23;73(5):1049–71. doi: 10.1007/s00267-024-01962-w (PMC11024003; doi:10.1007/s00267-024-01962-w)
Supplement: Supplementary file 1 — Supplementary Materials [file 267_2024_1962_MOESM1_ESM.pdf]

WILDLIFE CONSERVATION ON PRIVATE LAND: A SOCIAL-ECOLOGICAL SYSTEMS STUDY

Supplemental Material

This supplemental material presents partial dependency and variable importance plots based onRandom Forest analysis of mammal and bird richness, and focal mammal species: eastern barred bandicoot (Perameles gunnii), eastern quoll (Dasyurus viverrinus), long-nosed potoroo (Potorous tridactylus) and Tasmanian bettong (Bettongia gaimardi).

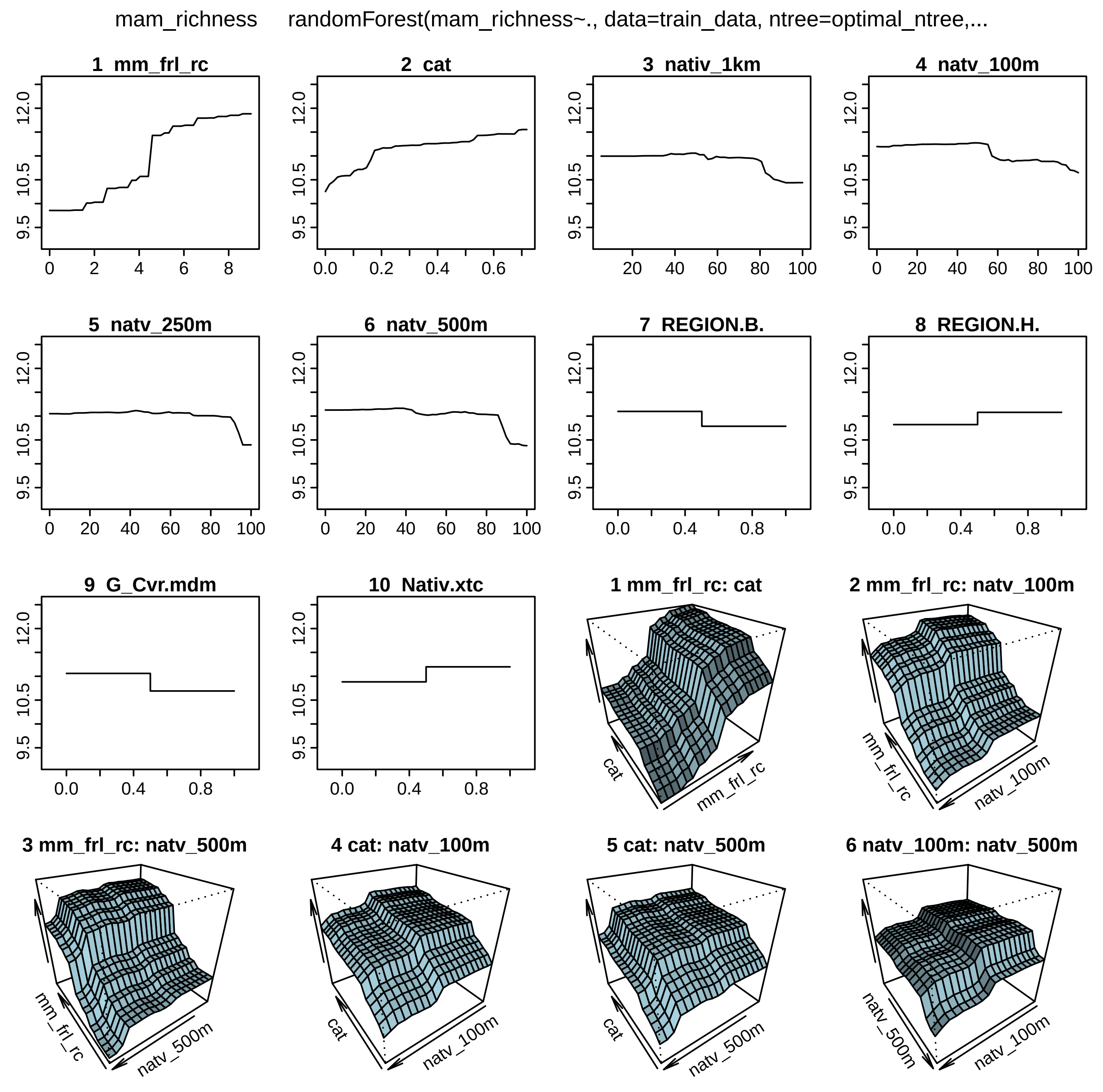
mam_richness randomForest(mam_richness~., data=train_data, ntree=optimal_ntree,...
1 mm_frl_rc
2 cat
3 nativ_1km
4 natv_100m
5 natv_250m
6 natv_500m
7 REGION.B.
8 REGION.H.
9 G_Cvr.mdm
10 Nativ.xtc
1 mm_frl_rc: cat
2 mm_frl_rc: natv_100m
3 mm_frl_rc: natv_500m
4 cat: natv_100m
5 cat: natv_500m
6 natv_100m: natv_500m

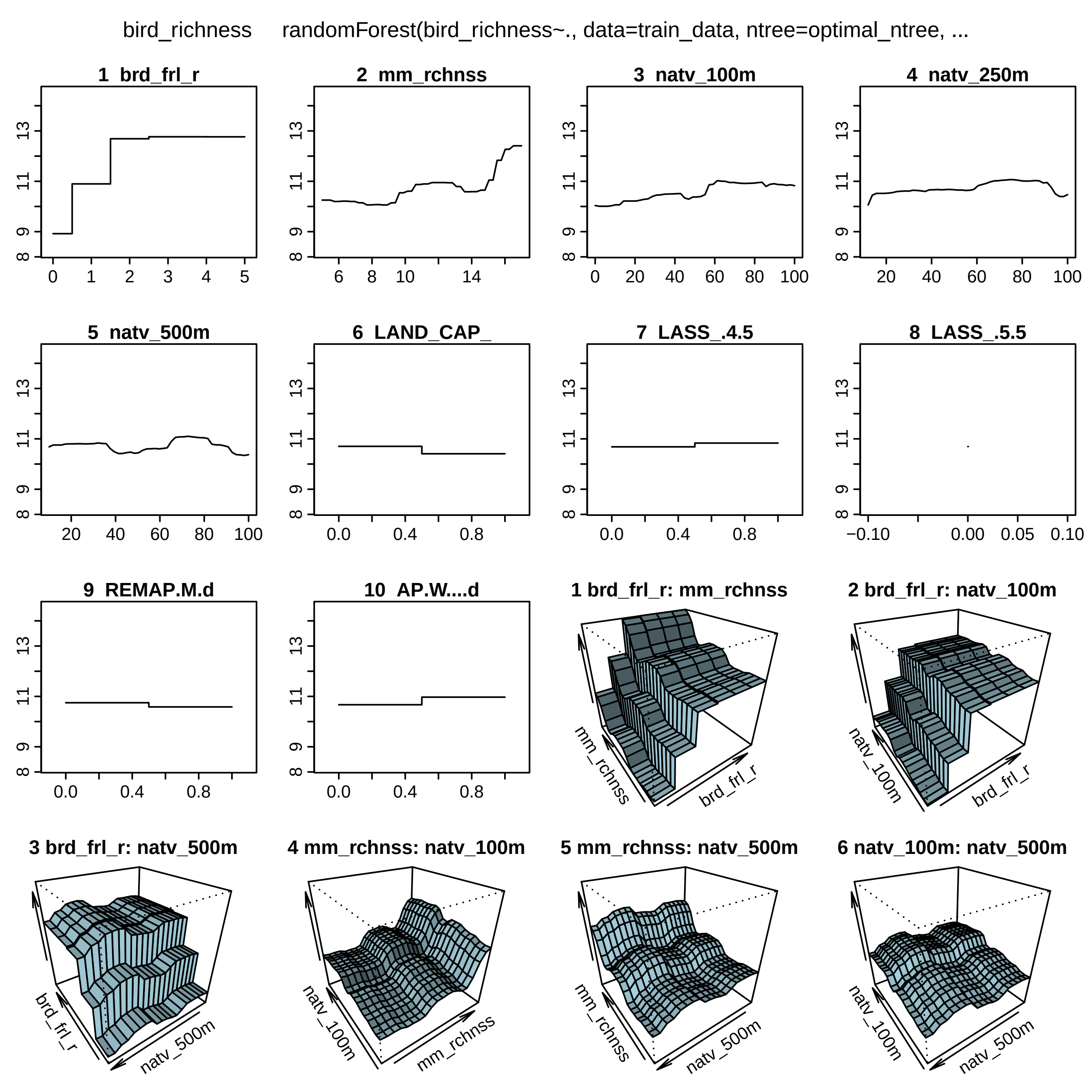

bird_richness randomForest(bird_richness~., data=train_data, ntree=optimal_ntree, ...
1 brd_frl_r
2 mm_rchnss
3 natv_100m
4 natv_250m
5 natv_500m
6 LAND_CAP_
7 LASS_.4.5
8 LASS_.5.5
9 REMAP.M.d
10 AP.W....d
1 brd_frl_r: mm_rchnss
2 brd_frl_r: natv_100m
3 brd_frl_r: natv_500m
4 mm_rchnss: natv_100m
5 mm_rchnss: natv_500m
6 natv_100m: natv_500m

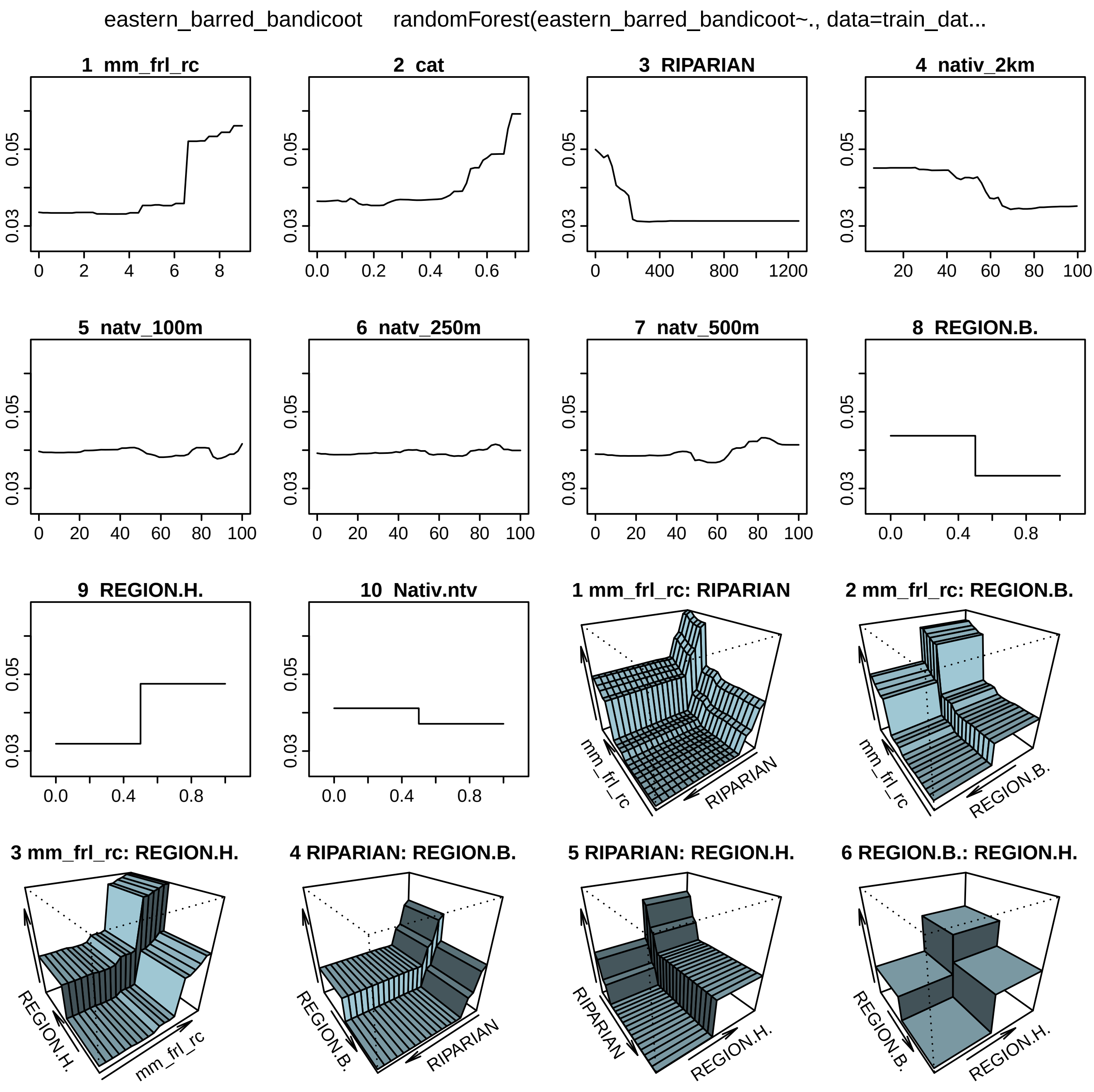
eastern_barred_bandicoot randomForest(eastern_barred_bandicoot~., data=train_dat...
1 mm_frl_rc
2 cat
3 RIPARIAN
4 nativ_2km
5 natv_100m
6 natv_250m
7 natv_500m
8 REGION.B.
9 REGION.H.
10 Nativ.ntv
1 mm_frl_rc: RIPARIAN
2 mm_frl_rc: REGION.B.
3 mm_frl_rc: REGION.H.
4 RIPARIAN: REGION.B.
5 RIPARIAN: REGION.H.
6 REGION.B.: REGION.H.
0.03
0.05
mm_frl_rc
RIPARIAN
REGION.B.
REGION.H.

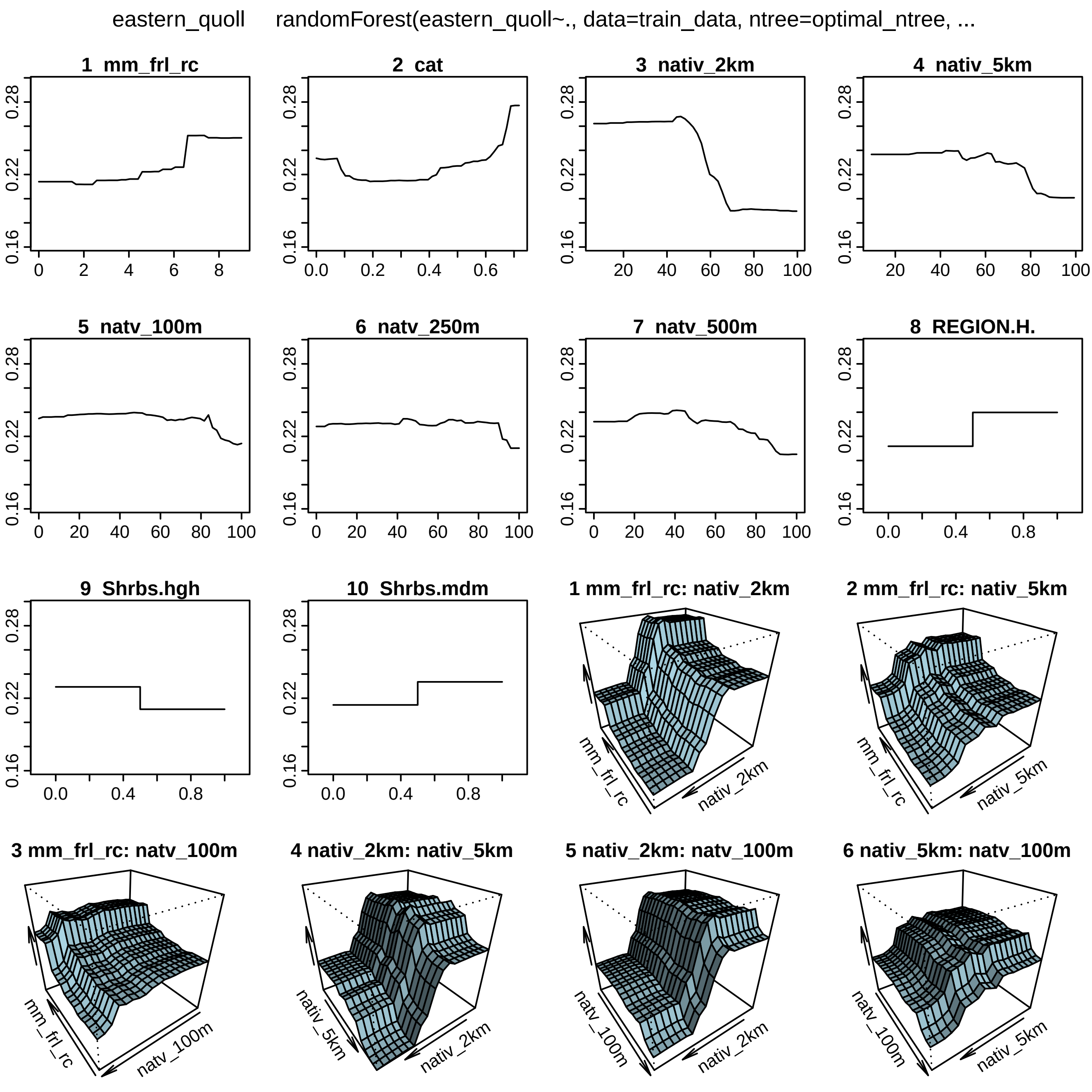
eastern_quoll randomForest(eastern_quoll~., data=train_data, ntree=optimal_ntree, ...
1 mm_frl_rc
2 cat
3 nativ_2km
4 nativ_5km
5 natv_100m
6 natv_250m
7 natv_500m
8 REGION.H.
9 Shrbs.hgh
10 Shrbs.mdm
1 mm_frl_rc: nativ_2km
2 mm_frl_rc: nativ_5km
3 mm_frl_rc: natv_100m
4 nativ_2km: nativ_5km
5 nativ_2km: natv_100m
6 nativ_5km: natv_100m
0.16
0.22
0.28
0 2 4 6 8
0.0 0.2 0.4 0.6
20 40 60 80 100
0 20 40 60 80 100
0.0 0.4 0.8
mm_frl_rc
nativ_2km
nativ_5km
natv_100m

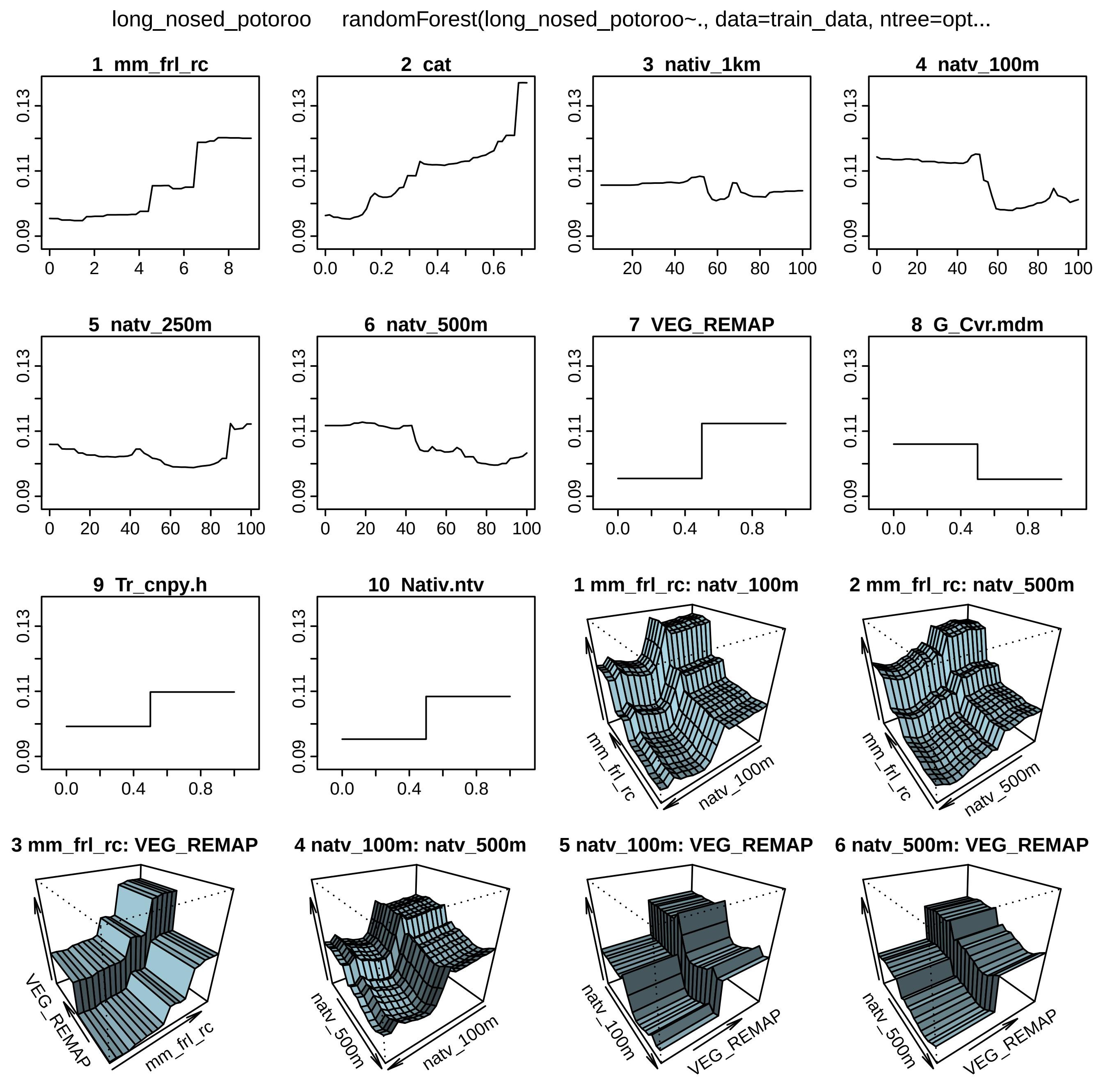

long_nosed_potoroo randomForest(long_nosed_potoroo~., data=train_data, ntree=opt...
1 mm_frl_rc
2 cat
3 nativ_1km
4 natv_100m
5 natv_250m
6 natv_500m
7 VEG_REMAP
8 G_Cvr.mdm
9 Tr_cnpy.h
10 Nativ.ntv
1 mm_frl_rc: natv_100m
2 mm_frl_rc: natv_500m
3 mm_frl_rc: VEG_REMAP
4 natv_100m: natv_500m
5 natv_100m: VEG_REMAP
6 natv_500m: VEG_REMAP
0.09
0.11
0.13

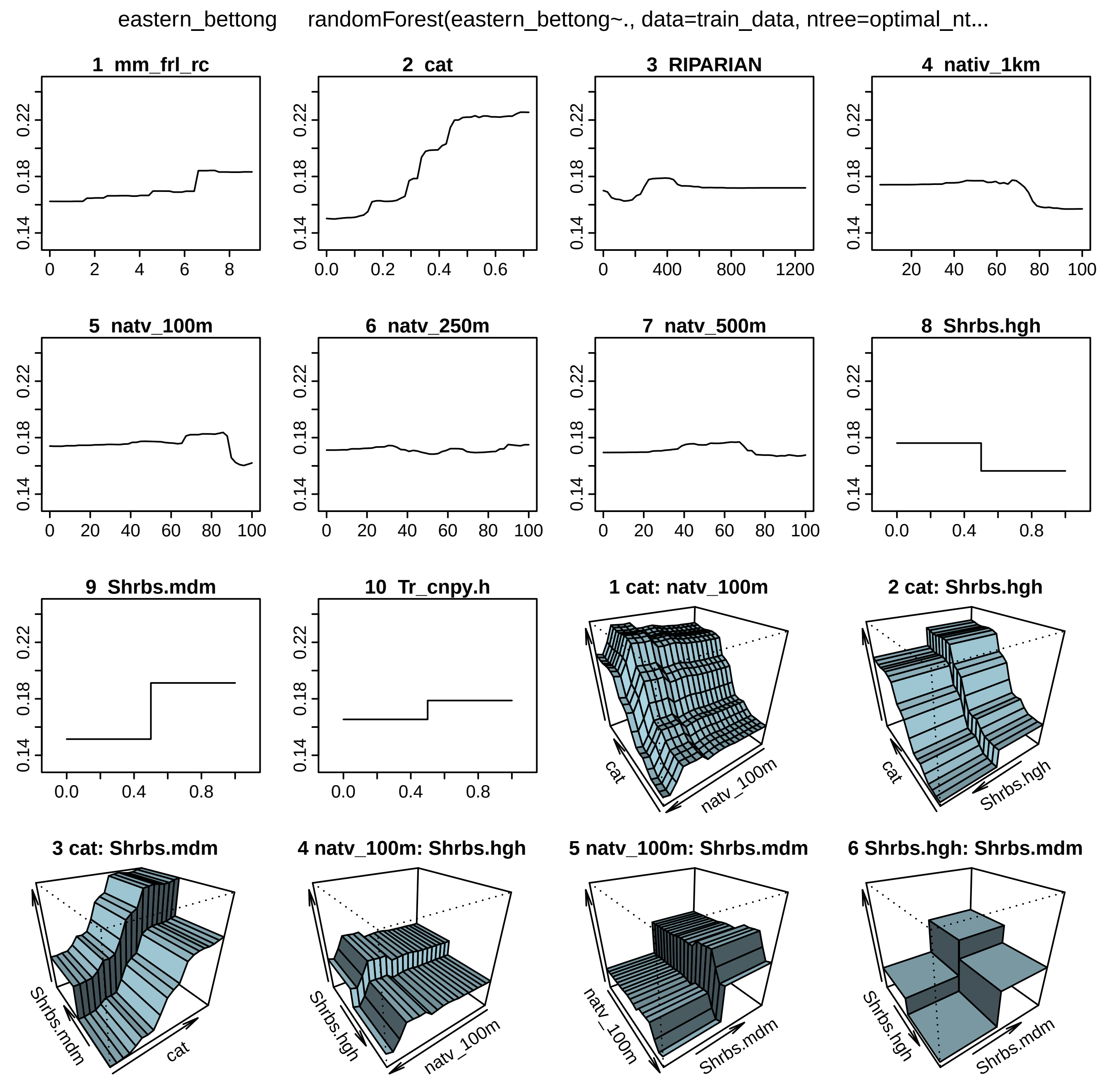
eastern_bettong randomForest(eastern_bettong~., data=train_data, ntree=optimal_nt...
1 mm_frl_rc
2 cat
3 RIPARIAN
4 nativ_1km
5 natv_100m
6 natv_250m
7 natv_500m
8 Shrbs.hgh
9 Shrbs.mdm
10 Tr_cnpy.h
1 cat: natv_100m
2 cat: Shrbs.hgh
3 cat: Shrbs.mdm
4 natv_100m: Shrbs.hgh
5 natv_100m: Shrbs.mdm
6 Shrbs.hgh: Shrbs.mdm
0.14
0.18
0.22
cat
natv_100m
Shrbs.hgh
Shrbs.mdm

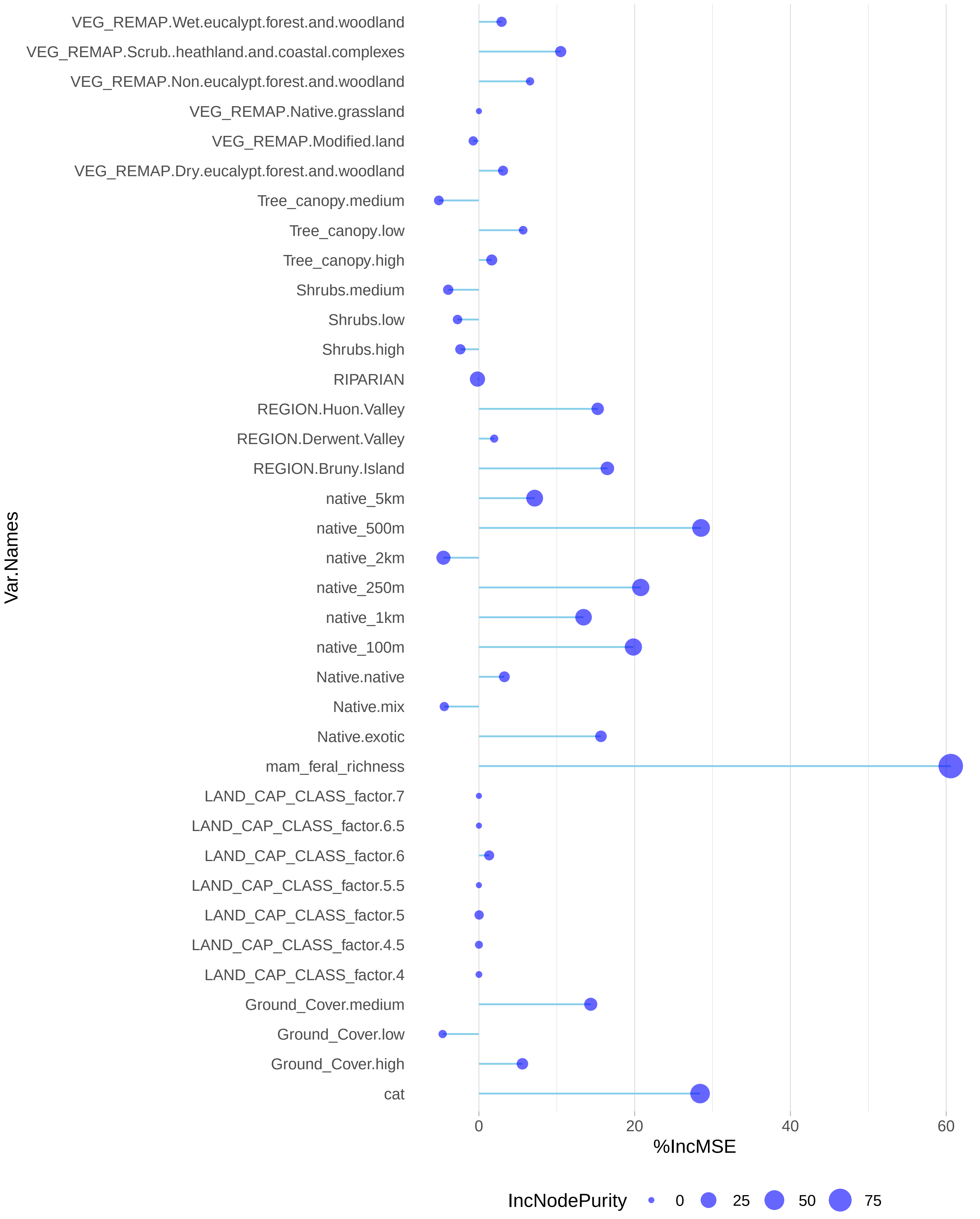

VEG_REMAP.Wet.eucalypt.forest.and.woodland
VEG_REMAP.Scrub..heathland.and.coastal.complexes
VEG_REMAP.Non.eucalypt.forest.and.woodland
VEG_REMAP.Native.grassland
VEG_REMAP.Modified.land
VEG_REMAP.Dry.eucalypt.forest.and.woodland
Tree_canopy.medium
Tree_canopy.low
Tree_canopy.high
Shrubs.medium
Shrubs.low
Shrubs.high
RIPARIAN
REGION.Huon.Valley
REGION.Derwent.Valley
REGION.Bruny.Island
native_5km
native_500m
native_2km
native_250m
native_1km
native_100m
Native.native
Native.mix
Native.exotic
mam_feral_richness
LAND_CAP_CLASS_factor.7
LAND_CAP_CLASS_factor.6.5
LAND_CAP_CLASS_factor.6
LAND_CAP_CLASS_factor.5.5
LAND_CAP_CLASS_factor.5
LAND_CAP_CLASS_factor.4.5
LAND_CAP_CLASS_factor.4
Ground_Cover.medium
Ground_Cover.low
Ground_Cover.high
cat
Var.Names
0
20
40
60
%IncMSE
IncNodePurity
0
25
50
75

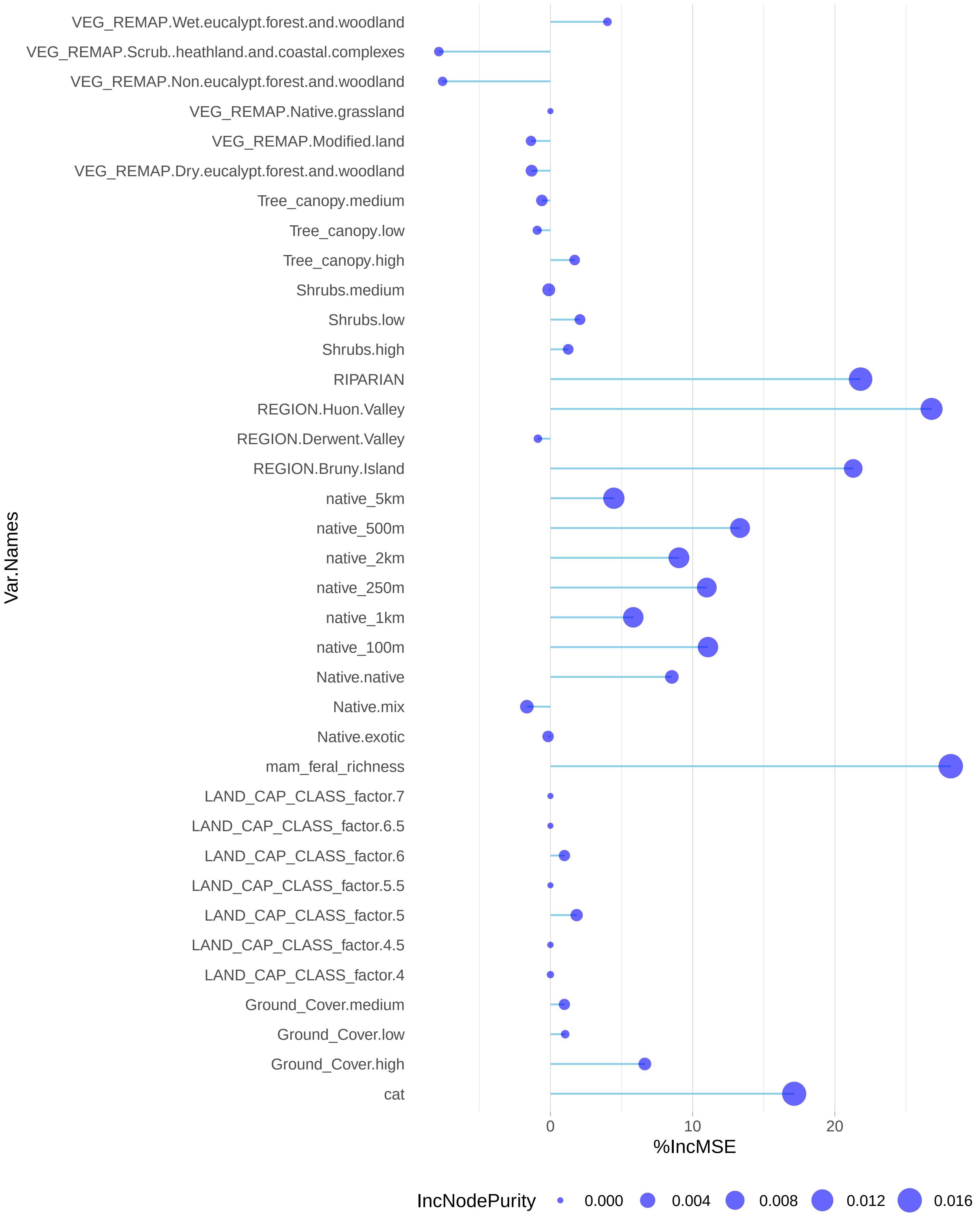

VEG_REMAP.Wet.eucalypt.forest.and.woodland
VEG_REMAP.Scrub..heathland.and.coastal.complexes
VEG_REMAP.Non.eucalypt.forest.and.woodland
VEG_REMAP.Native.grassland
VEG_REMAP.Modified.land
VEG_REMAP.Dry.eucalypt.forest.and.woodland
Tree_canopy.medium
Tree_canopy.low
Tree_canopy.high
Shrubs.medium
Shrubs.low
Shrubs.high
RIPARIAN
REGION.Huon.Valley
REGION.Derwent.Valley
REGION.Bruny.Island
native_5km
native_500m
native_2km
native_250m
native_1km
native_100m
Native.native
Native.mix
Native.exotic
mam_feral_richness
LAND_CAP_CLASS_factor.7
LAND_CAP_CLASS_factor.6.5
LAND_CAP_CLASS_factor.6
LAND_CAP_CLASS_factor.5.5
LAND_CAP_CLASS_factor.5
LAND_CAP_CLASS_factor.4.5
LAND_CAP_CLASS_factor.4
Ground_Cover.medium
Ground_Cover.low
Ground_Cover.high
cat
Var.Names
0
10
20
%IncMSE
IncNodePurity
0.000
0.004
0.008
0.012
0.016

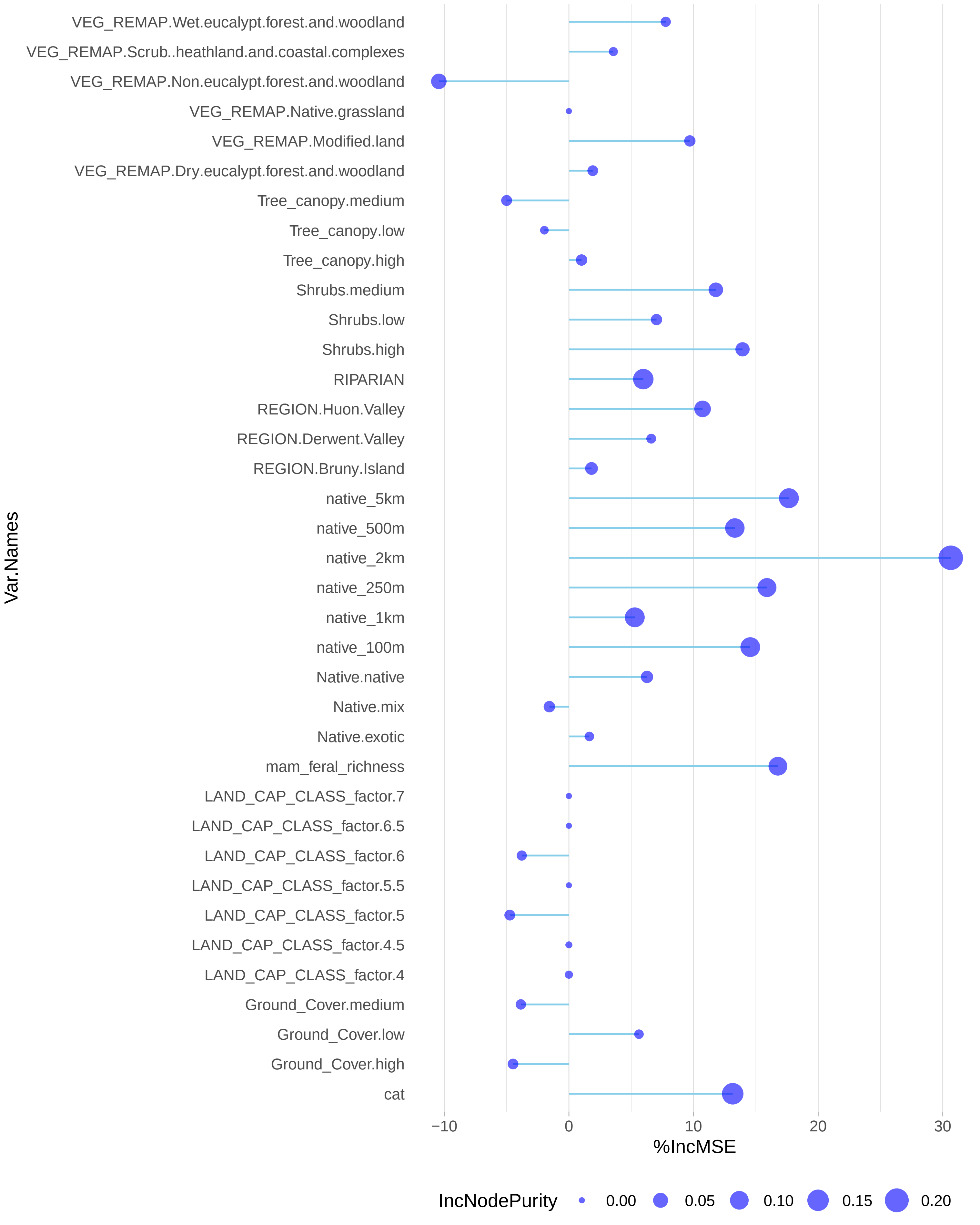
VEG_REMAP.Wet.eucalypt.forest.and.woodland
VEG_REMAP.Scrub..heathland.and.coastal.complexes
VEG_REMAP.Non.eucalypt.forest.and.woodland
VEG_REMAP.Native.grassland
VEG_REMAP.Modified.land
VEG_REMAP.Dry.eucalypt.forest.and.woodland
Tree_canopy.medium
Tree_canopy.low
Tree_canopy.high
Shrubs.medium
Shrubs.low
Shrubs.high
RIPARIAN
REGION.Huon.Valley
REGION.Derwent.Valley
REGION.Bruny.Island
native_5km
native_500m
native_2km
native_250m
native_1km
native_100m
Native.native
Native.mix
Native.exotic
mam_feral_richness
LAND_CAP_CLASS_factor.7
LAND_CAP_CLASS_factor.6.5
LAND_CAP_CLASS_factor.6
LAND_CAP_CLASS_factor.5.5
LAND_CAP_CLASS_factor.5
LAND_CAP_CLASS_factor.4.5
LAND_CAP_CLASS_factor.4
Ground_Cover.medium
Ground_Cover.low
Ground_Cover.high
cat
Var.Names
−10
0
10
20
30
%IncMSE
IncNodePurity
0.00
0.05
0.10
0.15
0.20

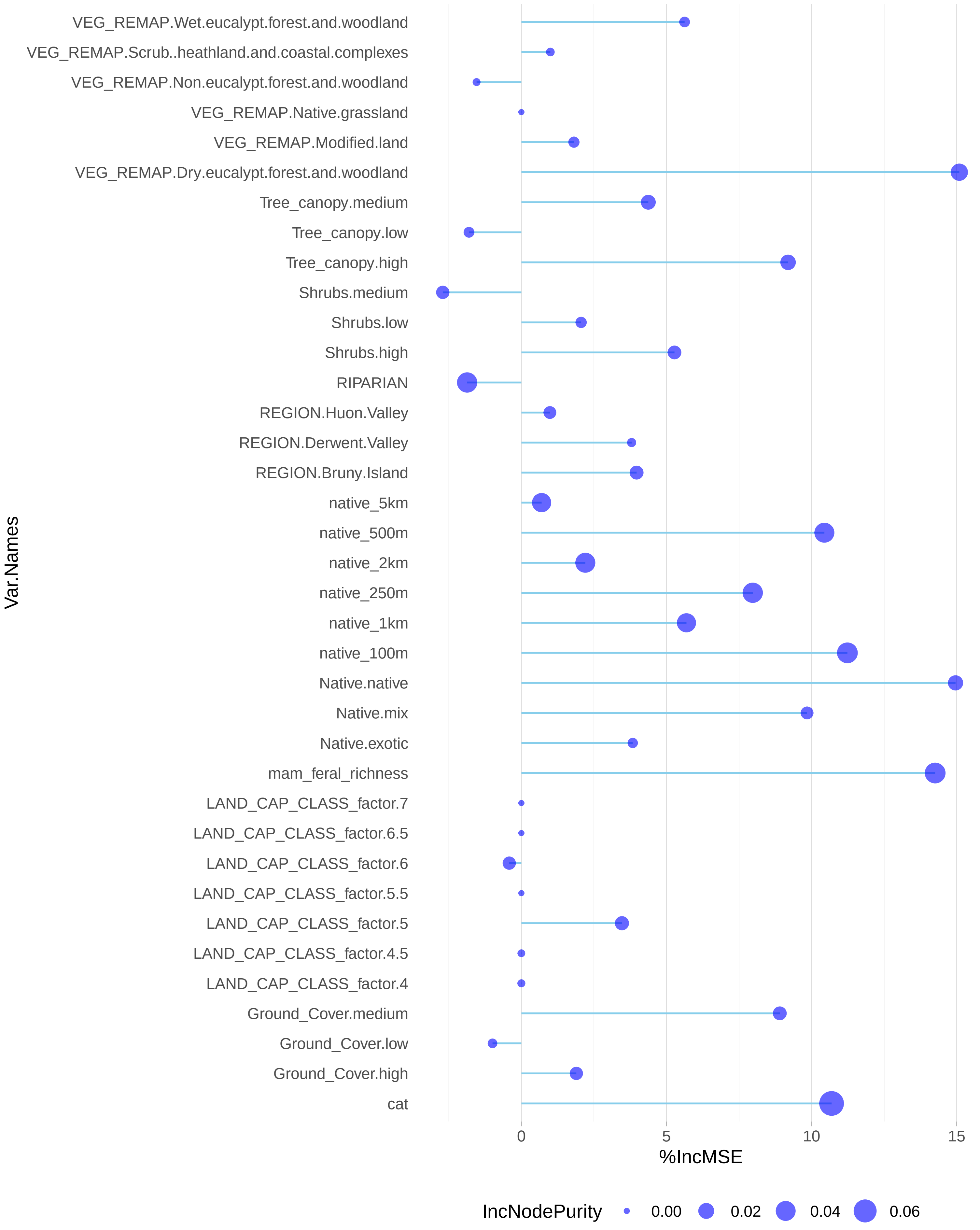
VEG_REMAP.Wet.eucalypt.forest.and.woodland
VEG_REMAP.Scrub..heathland.and.coastal.complexes
VEG_REMAP.Non.eucalypt.forest.and.woodland
VEG_REMAP.Native.grassland
VEG_REMAP.Modified.land
VEG_REMAP.Dry.eucalypt.forest.and.woodland
Tree_canopy.medium
Tree_canopy.low
Tree_canopy.high
Shrubs.medium
Shrubs.low
Shrubs.high
RIPARIAN
REGION.Huon.Valley
REGION.Derwent.Valley
REGION.Bruny.Island
native_5km
native_500m
native_2km
native_250m
native_1km
native_100m
Native.native
Native.mix
Native.exotic
mam_feral_richness
LAND_CAP_CLASS_factor.7
LAND_CAP_CLASS_factor.6.5
LAND_CAP_CLASS_factor.6
LAND_CAP_CLASS_factor.5.5
LAND_CAP_CLASS_factor.5
LAND_CAP_CLASS_factor.4.5
LAND_CAP_CLASS_factor.4
Ground_Cover.medium
Ground_Cover.low
Ground_Cover.high
cat
Var.Names
0
5
10
15
%IncMSE
IncNodePurity
0.00
0.02
0.04
0.06

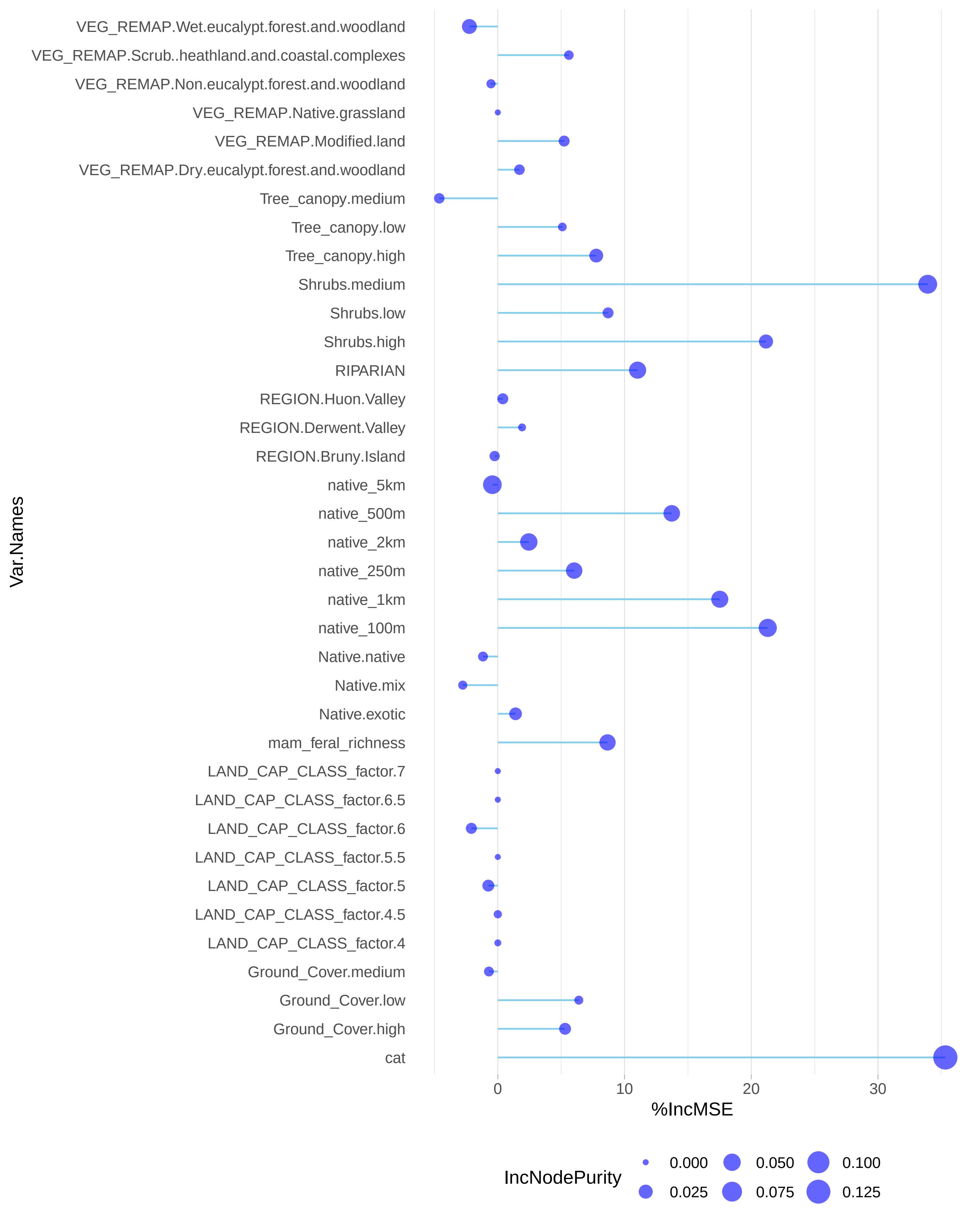
VEG_REMAP.Wet.eucalypt.forest.and.woodland
VEG_REMAP.Scrub..heathland.and.coastal.complexes
VEG_REMAP.Non.eucalypt.forest.and.woodland
VEG_REMAP.Native.grassland
VEG_REMAP.Modified.land
VEG_REMAP.Dry.eucalypt.forest.and.woodland
Tree_canopy.medium
Tree_canopy.low
Tree_canopy.high
Shrubs.medium
Shrubs.low
Shrubs.high
RIPARIAN
REGION.Huon.Valley
REGION.Derwent.Valley
REGION.Bruny.Island
native_5km
native_500m
native_2km
native_250m
native_1km
native_100m
Native.native
Native.mix
Native.exotic
mam_feral_richness
LAND_CAP_CLASS_factor.7
LAND_CAP_CLASS_factor.6.5
LAND_CAP_CLASS_factor.6
LAND_CAP_CLASS_factor.5.5
LAND_CAP_CLASS_factor.5
LAND_CAP_CLASS_factor.4.5
LAND_CAP_CLASS_factor.4
Ground_Cover.medium
Ground_Cover.low
Ground_Cover.high
cat
Var.Names
0
10
20
30
%IncMSE
IncNodePurity
0.000
0.025
0.050
0.075
0.100
0.125
